# Partitioning of the truncated insulin receptor DAF-2B between homodimers and heterodimers influences insulin signaling in *C. elegans*

**DOI:** 10.1371/journal.pgen.1012240

**Published:** 2026-07-23

**Authors:** Bryan A. Martinez, Anne M. Stene, Jonathan I. Hauser, Karla J. Opperman, Jonathan N. Sachs, Anthony R. Braun, Matthew S. Gill

**Affiliations:** 1 Masonic Institute on the Biology of Aging and Metabolism, University of Minnesota, Minneapolis, Minnesota, United States of America; 2 Department of Genetics, Cell Biology, and Development, University of Minnesota Medical School, Minneapolis, Minnesota, United States of America; 3 Department of Biomedical Engineering, University of Minnesota, Minneapolis, Minnesota, United States of America; UT Austin: The University of Texas at Austin, UNITED STATES OF AMERICA

## Abstract

Insulin / insulin-like growth factor signaling (IIS) in *C. elegans* is mediated by the DAF-2 receptor and insulin-like peptides (ILP) that can act as agonists or antagonists. DAF-2 signaling is also affected by DAF-2B, a truncated, non-signaling, secreted isoform of DAF-2 that acts as a decoy receptor by sequestering ILPs. In this study, we performed a forward genetic screen for modifiers of DAF-2B protein expression and identified a mutation in *unc-31* that increased DAF-2B*.* UNC-31 is involved in dense core vesicle docking and is required for neuropeptide secretion, including ILPs. As a result, *unc-31* mutants constitutively enter the dauer larval stage and are long-lived due to reduced IIS. We find that increased nervous system DAF-2B accumulation is associated with reduced agonist ILP availability, in both *unc-31* mutants and wild type worms. Using auxin-induced degradation (AID) and fluorescence lifetime imaging microscopy-Förster resonance energy transfer (FLIM-FRET), we find that a significant fraction of nervous system DAF-2B is in the form of a heterodimeric complex with a full-length DAF-2 receptor isoform, representing a new class of DAF-2 hybrid receptor. In *unc-31* mutants, DAF-2B also undergoes endocytosis in neurons in an AP2-dependent manner and genetic manipulation of *daf-2b* in the *unc-31* mutant suggests that DAF-2B homodimers may function to reinforce a reduced insulin signaling state by clearing agonist ILPs from the extracellular space. These findings indicate that DAF-2B not only forms homodimers, but also hybrid receptors with full-length DAF-2, to regulate the activity of the large and diverse family of ILPs in *C. elegans*.

## Author summary

In the nematode *C. elegans*, the insulin-like signaling pathway influences development and aging through a receptor called DAF-2 that has similarity to mammalian insulin and insulin-like growth factor receptors. The existence of multiple DAF-2 receptor isoforms, as well as agonist and antagonist insulin-like peptide ligands, generates considerable signaling complexity. We have previously identified a non-signaling, truncated receptor isoform called DAF-2B that dimerizes with itself and regulates the interaction of ILP ligands with the full-length signaling isoform of the DAF-2 receptor. Here we demonstrate that DAF-2B can also dimerize with full-length DAF-2 receptor isoforms to generate hybrid receptors and the balance between DAF-2B self-dimers and hybrid receptors influences insulin-like signaling. Differential dimerization of DAF-2 receptor isoforms provides a new mechanism to explain how the nematode maintains temporal and spatial regulation of insulin signaling in the context of multiple agonist and antagonist ILPs.

## Introduction

**Fig 1:**
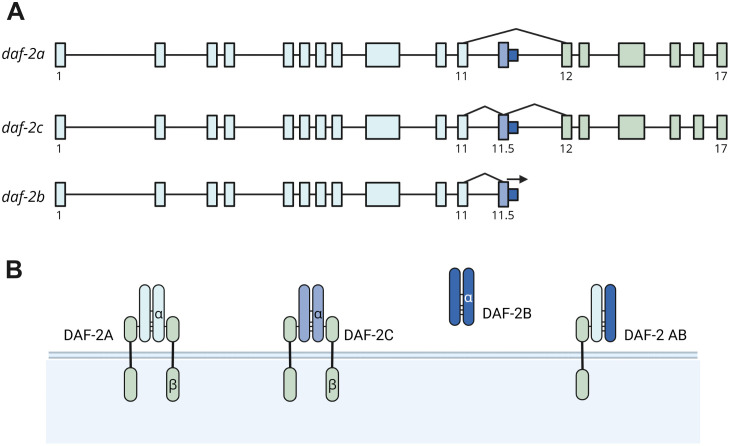
Alternative splicing generates multiple DAF-2 isoforms. **(A)** Alternative splicing generates *daf-2a, daf-2c* and *daf-2b* transcripts. Splicing between exon 11 and exon 12 generates *daf-2a,* whereas *daf-2c* includes the cassette exon 11.5. *daf-2b* is generated by intronic polyadenylation and lacks exons 12–17. Blue exons encode the α subunit and green exons encode the β subunit. **(B)** DAF-2 isoforms form dimers linked by disulfide bonds. The α subunit forms the extracellular ligand binding domain (blue) and the β subunit contains the transmembrane domain and the tyrosine kinase signaling domain (green). DAF-2A and DAF-2C are membrane bound homodimers, while DAF-2B is a secreted homodimer since it lacks the β subunit. The potential for DAF-2AB heterodimers has been demonstrated in cell culture transfection studies [5] and other dimer configurations are theoretically possible. Created in BioRender. Gill, M. (2026) https://BioRender.com/5hynawv.

In the nematode *C. elegans*, the insulin / insulin-like growth factor signaling (IIS) pathway influences growth, metabolism, stress responses and longevity via secretion of insulin-like peptides (ILPs) that target the insulin/IGF-I receptor homolog, DAF-2 [1]. Signaling complexity arises from multiple ILPs [2–4] and the expression of several DAF-2 isoforms that arise from alternative splicing [5,6] (Fig 1A). Full-length *daf-2a* and *daf-2c* transcripts generate signaling-competent proteins that contain an α subunit (ligand binding domain) and a β subunit (transmembrane and intracellular tyrosine kinase signaling domains) (Fig 1B). DAF-2A and DAF-2C differ by the absence or presence, respectively, of a short peptide sequence on the C-terminus of the α subunit that is encoded by the alternatively spliced exon 11.5 [6] (Fig 1). Mature DAF-2 receptor complexes are covalently linked dimers due to the presence of disulfide bonds that are formed between the α subunits in the endoplasmic reticulum (ER) when the proteins are being processed for secretion.

Full-length DAF-2 receptors are predicted to bind and respond to ILPs, of which there are 40 in the nematode genome, that exhibit varying degrees of homology with mammalian insulin, IGFs and ILPs [2–4]. Expression studies indicate that *C. elegans* ILPs differ in their temporal and spatial expression [7,8], and groups of ILPs influence specific phenotypes by operating in local circuits [9], as well as signaling between different tissues [10,11]. Interestingly, nematode ILPs can act as agonists or antagonists of IIS, while some can function as both, depending on the context [4]. For example, DAF-28 is considered an agonist ILP, as it consistently promotes IIS [3,12]. On the other hand, INS-18 can be an antagonist ILP, as it reduces IIS to promote entry into the dauer diapause stage [13,14], but it can have opposite effects on IIS in other contexts [9]. It is important to note that these designations are based on phenotypic outcomes rather than changes in the biochemical activity of the receptor. To date, direct binding of ILPs to DAF-2 has not been demonstrated either *in vitro* or *in vivo,* although purified INS-6 has been shown to activate the human insulin receptor [15].

We have previously characterized another alternatively spliced DAF-2 isoform, DAF-2B, that retains the ligand-binding α subunit, and therefore forms homodimers, but lacks the β subunit [5] (Fig 1). Consequently, DAF-2B homodimers are not anchored in the plasma membrane and do not signal like full-length receptors but are instead secreted into the extracellular environment. Genetic interaction studies suggest that DAF-2B acts as a decoy receptor and regulates IIS phenotypes by sequestration of ILPs away from full length receptors [5]. Cell culture transfection experiments with DAF-2A and DAF-2B, not only confirmed the existence of covalently linked DAF-2A homodimers and DAF-2B homodimers but also demonstrated that DAF-2A / DAF-2B heterodimers could be generated, whereby covalent disulfide bonds form between the DAF-2A and DAF-2B α subunits [5]. These heterodimers are expected to localize to the cell surface by virtue of the DAF-2A transmembrane domain and could represent another class of decoy receptor (Fig 1B). However, the existence of these heterodimers *in vivo* and their physiological relevance has not been demonstrated.

We have recently shown that the splicing factor RSP-2 influences DAF-2B expression [16], but beyond this, little is known about the factors that regulate DAF-2B levels or activity. In this study, we performed a forward mutagenesis screen using a DAF-2B::mScarlet translational reporter strain and discovered that mutations in *unc-31* lead to elevated neuronal DAF-2B. UNC-31 is involved in neuropeptide secretion via docking of dense core vesicles [17–20] and has been implicated in secretion of neuronal ILPs [21,22]. We provide evidence that the neuronal accumulation of DAF-2B in *unc-31* mutants is a response to reduced agonist ILP secretion and the corresponding reduction in IIS. We also find that neuronal DAF-2B is partitioned between DAF-2B homodimers and cell surface heterodimers with full-length DAF-2, the latter representing a new class of DAF-2 hybrid receptor that may have altered affinity for ILPs compared with DAF-2B homodimers.

## Results

### Mutations in *unc-31* increase DAF-2B::mScarlet levels in the nervous system

**Fig 2:**
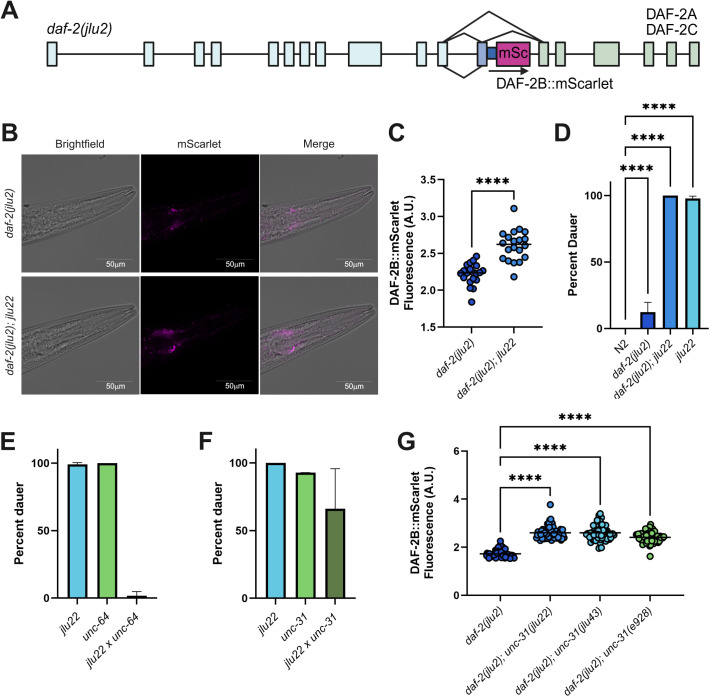
Mutations in *unc-31* increase DAF-2B::mScarlet levels in the nervous system. **(A)** In *daf-2(jlu2),* the mScarlet coding sequence was inserted at the end of the DAF-2B-specific genomic sequence to generate a DAF-2B::mScarlet translational reporter [5]. The diagram was created in BioRender. Gill, M. (2026) https://BioRender.com/5hynawv. **(B)** Representative images illustrating neuronal DAF-2B::mScarlet expression in *daf-2(jlu2)* control and *daf-2(jlu2); jlu22* mutant young adult worms. **(C)** DAF-2B::mScarlet is significantly elevated in the nervous system of *daf-2(jlu2); jlu22* mutants compared with *daf-2(jlu2)* control animals. Student’s *t*-test **** p < 0.0001. **(D)** *jlu22* mutants form dauers at 27°C in both the *daf-2(jlu2)* and wild type backgrounds. Data are pooled from 4 biological replicates, with a total of 20 populations per condition. One-way ANOVA with Dunnett’s post-hoc test for pair-wise comparison vs N2 control, **** p < 0.0001. **(E)** *jlu22* complements *unc-64(e246)* with respect to dauer formation at 27°C. **(F)** *jlu22* fails to complement *unc-31(e928)* with respect to dauer formation at 27°C. **(G)** Neuronal DAF-2B::mScarlet expression is elevated in young adult worms in multiple *unc-31* alleles. *unc-31(jlu22)* is the 10849G > A isolate from the screen; *unc-31(jlu43)* is the 10849G > A mutation generated by CRISPR; *unc-31(e928)* is a null allele. Data are pooled from 3 biological replicates. One-way ANOVA with Dunnett’s post-hoc test for pair-wise comparison vs *daf-2(jlu2)* control, **** p < 0.0001.

To identify new regulators of DAF-2B expression, we performed ethyl methanesulfonate (EMS) mutagenesis in the *daf-2(jlu2)* background, which has an insertion of mScarlet in frame with the unique *daf-2b* C-terminal genomic sequence (DAF-2B::mScarlet, Fig 2A) [5]. After mutagenesis, adult F2 progeny were screened for changes in fluorescence intensity in the nervous system*.* One isolate, *daf-2(jlu2)*; *jlu22,* had significantly elevated nervous system DAF-2B::mScarlet expression and was chosen for further characterization (Fig 2B and 2C). In addition to elevated DAF-2B::mScarlet, we noted that *daf-2(jlu2); jlu22* mutants were uncoordinated (Unc) and constitutively formed dauers (Daf-c) at high temperatures (Fig 2D), phenotypes that are characteristic of *unc-31* and *unc-64* loss-of-function alleles [21,23–25]. We therefore hypothesized that the *daf-2(jlu2); jlu22* mutant carried a mutation in either *unc-31* or *unc-64*. To test this, we performed complementation assays of *jlu22* against *unc-31(e928)* and *unc-64(e246)*, with respect to the Daf-c phenotype. First, we crossed *jlu22* away from *daf-2(jlu2)* and confirmed that the mutants still formed dauers at high temperatures (Fig 2D). Complementation was performed with *jlu22* hermaphrodites and heterozygous *unc-31(e928)* and *unc-64(e246)* males, since Unc males do not mate well. Thus, if the alleles failed to complement, the distribution of mutant phenotypes would be 50%. Correspondingly, we found that *jlu22* complemented *unc-64(e246)* (Fig 2E) but failed to complement *unc-31(e928)* (Fig 2F). Therefore, *jlu22* carries a loss-of-function mutation in *unc-31* and was designated *unc-31(jlu22)*.

To characterize the mutation in *unc-31(jlu22)* animals, we performed whole genome sequencing and identified two mutations in the *unc-31* locus. The first, a base pair substitution in intron 9 (7219A > C), corresponds to a naturally occurring polymorphism (WBVar01274649 [26]). The second, a base pair substitution (10849G > A) in exon 15, is predicted to generate a premature stop codon (S1 Fig). To confirm this as the causal mutation, we used CRISPR / Cas9 genome editing to introduce the 10849G > A mutation into the *daf-2(jlu2)* background, generating *daf-2(jlu2); unc-31(jlu43),* and observed a similar increase in neuronal DAF-2B::mScarlet levels to that seen in the original *daf-2(jlu2); unc-31(jlu22)* isolate (Figs 2G and S1). We also crossed *daf-2(jlu2)* into the *unc-31(e928)* null mutant background and found that *unc-31(e928)* phenocopies *unc-31(jlu22)* and *unc-31(jlu43)* with respect to elevated DAF-2B::mScarlet (Figs 2G and S1). We therefore conclude that *unc-31* loss-of-function was the genetic cause of elevated DAF-2B::mScarlet fluorescence in the nervous system. For subsequent experiments we used the *unc-31(e928)* null allele. We note that since mScarlet fluorescence is sensitive to pH and many subcellular compartments differ in their pH levels, a change in DAF-2B::mScarlet intensity could be due to changes in protein abundance and / or changes in cellular compartmentalization.

### DAF-2B secretion is elevated in *unc-31* animals

**Fig 3:**
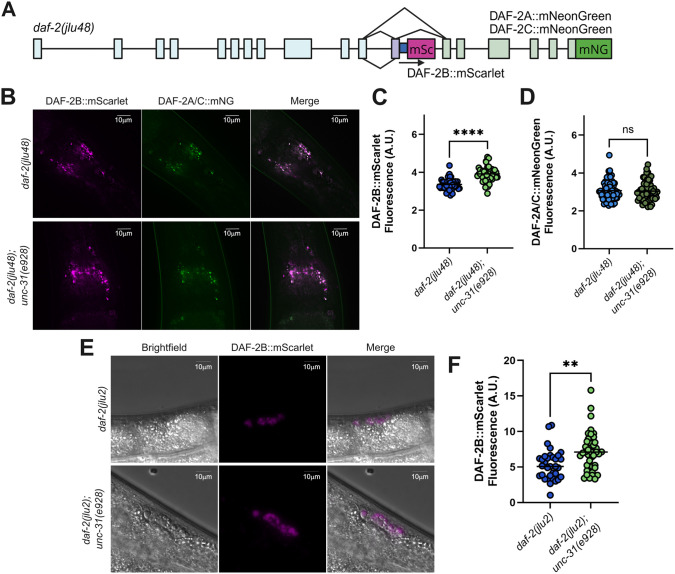
DAF-2B secretion is elevated in *unc-31* mutants. **(A)** In *daf-2(jlu48),* the mScarlet coding sequence was inserted at the end of the DAF-2B-specific sequence in a background in which mNeonGreen had already been inserted after exon 17, generating a DAF-2B::mScarlet and DAF-2A/C::mNeonGreen a translational reporter from the genomic locus. The diagram was created in BioRender. Gill, M. (2026) https://BioRender.com/5hynawv. **(B)** Representative images showing expression of DAF-2B::mScarlet and DAF-2A/C::mNeonGreen in the nervous system of wild type and *unc-31(e928)* day 1 adults. **(C)** Neuronal DAF-2B mScarlet is elevated in *daf-2(jlu48); unc-31(e928)* mutants compared with *daf-2(jlu48)* controls. **(D)** Neuronal DAF-2A/C::mNeonGreen is not different between *daf-2(jlu48)* and *daf-2(jlu48); unc-31(e928)* animals. **(E)** Representative images illustrating accumulation of DAF-2B::mScarlet in coelomocytes of *daf-2(jlu2)* control and *daf-2(jlu2); unc-31(e928)* mutant animals. **(F)** DAF-2B::mScarlet accumulation in coelomocytes is significantly elevated in *daf-2(jlu2); unc-31(e928)* mutants compared with *daf-2(jlu2)* controls. For C, D & F, data are pooled from 3 biological replicates. Pair-wise comparisons: Student’s *t*-test, ns = not significant, ** p < 0.01, ****p < 0.0001.

In addition to elevated DAF-2B::mScarlet fluorescence in the nervous system, we found that there was a significant increase in *daf-2b* mRNA in *unc-31(e928)* mutants, as well as increased levels of *daf-2a* and *daf-2c* transcripts (S2 Fig). To determine whether DAF-2A and DAF-2C proteins were also elevated in the nervous system, we inserted mScarlet into the *daf-2b* genomic locus of *daf-2(hq61),* a background in which mNeonGreen had already been inserted after exon 17 to produce a translational reporter for DAF-2A and DAF-2C [27] (Fig 3A). The resulting strain, *daf-2(jlu48[daf-2b::mScarlet daf-2a/c::mNeonGreen])* was then crossed into the *unc-31(e928)* mutant background. There was considerable co-localization of DAF-2B::mScarlet and DAF-2A/C::mNeonGreen in the nervous system of both wild type and *unc-31* mutants (Fig 3B). As expected, nervous system DAF-2B::mScarlet was significantly elevated in *daf-2(jlu48); unc-31(e928)* compared with controls (Fig 3C), while nervous system DAF-2A/C::mNeonGreen expression was not significantly different (Fig 3D). This suggests that DAF-2A/C protein levels may be upregulated in other non-neuronal tissues.

UNC-31 is orthologous to the human protein CADPS (calcium dependent secretion activator), also known as CAPS (calcium dependent activator protein for secretion) [17]. In *C. elegans,* this protein acts as a docking site and tether for dense core vesicles (DCVs) and a facilitator for SNARE complex assembly [19,20]. Therefore, a possible explanation for elevated neuronal DAF-2B::mScarlet in *unc-31* animals is that DAF-2B is packaged into DCVs and their defective release leads to intracellular accumulation of DAF-2B positive vesicles and reduced DAF-2B secretion. However, in *unc-31(e928)* mutants, we observed increased levels of DAF-2B::mScarlet in coelomocytes (Fig 3E and 3F), macrophage-like cells that internalize extracellular material by endocytosis [28]. Accumulation of fluorescent protein in coelomocytes is a well-established marker of secretion in *C. elegans* [29] and thus elevated DAF-2B::mScarlet in these cells in *unc-31(e928)* animals indicates that secretion of DAF-2B is not impaired but is elevated.

### Increased nervous system DAF-2B is associated with reduced IIS

**Fig 4:**
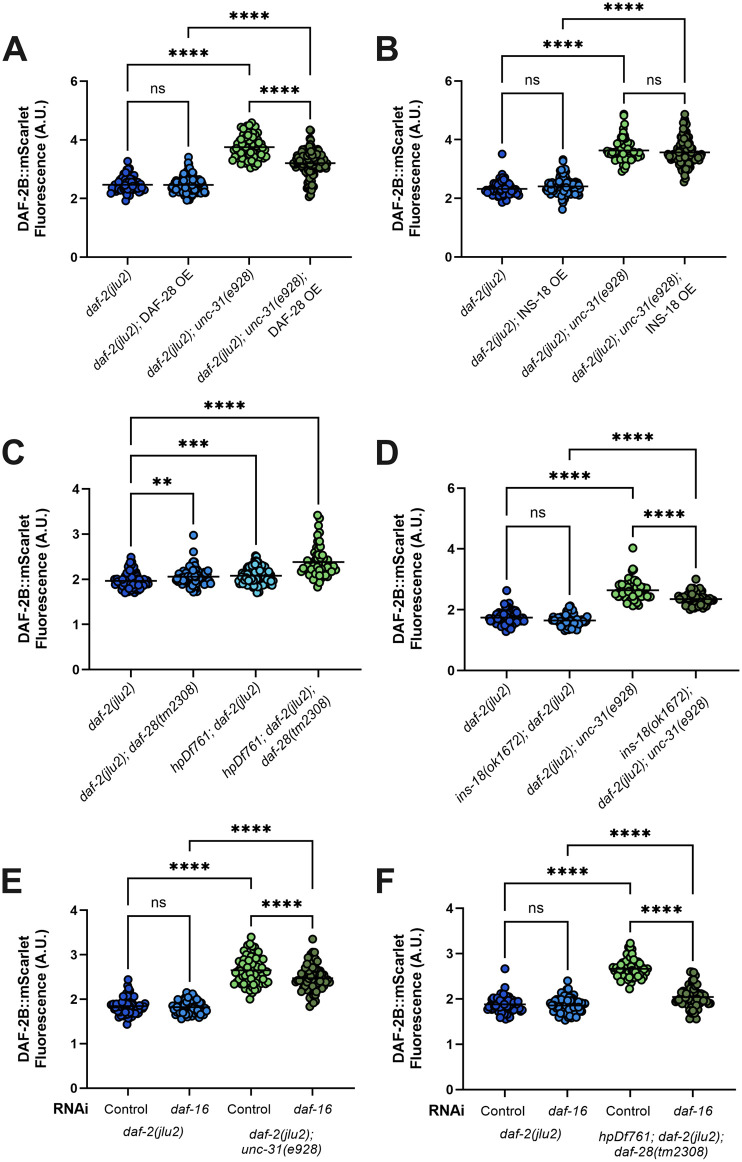
Increased DAF-2B is associated with reduced IIS. **(A)** Overexpression (OE) of the ILP agonist DAF-28 decreases nervous system DAF-2B::mScarlet expression in *unc-31(e928)* mutants but not in wild type. **(B)** INS-18 OE does not affect levels of nervous system DAF-2B::mScarlet in wild type or *unc-31(e928)* mutants. **(C)** Deletion of multiple agonist ILPs increases expression of DAF-2B::mScarlet in a wild type background. *hpDf761* is a combined deletion of *ins-4, ins-5* and *ins-6.* **(D)** Deletion of the ILP antagonist *ins-18* decreases nervous system DAF-2B::mScarlet expression in *unc-31(e928)* mutants, but not wild type. **(E)** RNAi of *daf-16* reduces expression of nervous system DAF-2B::mScarlet in *unc-31(e928)* mutants. **(F)** RNAi of *daf-16* reduces expression of nervous system DAF-2B::mScarlet in a multiple ILP agonist deletion background. *hpDf761; daf-2(jlu2); daf-28(tm2308)* animals on control RNAi exhibited 100% dauer arrest but spontaneously recovered within 24 h. We therefore imaged these recovered animals as D1 adults. In contrast, *daf-16* RNAi fully suppressed the Daf-c phenotype and these animals were imaged as D1 adults. Data are pooled from 3 biological replicates, except (C) (4 replicates). One-way ANOVA with Sidak’s post-hoc test for indicated pair-wise comparisons: ns = not significant, ** p < 0.01, *** p < 0.001, **** p < 0.0001.

Mutant *unc-31* animals are broadly defective in neuropeptide secretion and exhibit multiple phenotypes, including extended longevity and constitutive dauer formation at high temperatures [21,30], which are associated with reduced ILP secretion and IIS. We therefore hypothesized that alterations in ILP secretion may be responsible for increased nervous system DAF-2B levels in *unc-31* mutants. DAF-28 is an ILP agonist which suppresses dauer formation and L1 starvation survival when overexpressed [31]. In adult wild type animals, DAF-28 overexpression (OE) had no effect on nervous system DAF-2B::mScarlet levels, but DAF-28 OE in the *unc-31* mutant reduced nervous system DAF-2B::mScarlet (Fig 4A). In contrast, INS-18 can act as an antagonist ILP because loss-of-function mutations in *ins-18* reduce dauer formation [32] and reduce lifespan in *daf-2* mutant backgrounds [13]. However, INS-18 OE did not change nervous system DAF-2B levels in either wild type or *unc-31* mutant adults (Fig 4B).

We next examined whether a reduction in ILP agonists alone can lead to increased nervous system DAF-2B::mScarlet. Since single agonist ILP deletions have modest effects on IIS phenotypes, we crossed *daf-2(jlu2)* with *hpDf761; daf-28(tm2308)*, where *hpDf761* is a complex rearrangement that knocks out several ILP agonists together (*ins-4, ins-5,* and *ins-6*). From this cross, we also derived *daf-2(jlu2)*; *daf-28(tm2308)*, as well as *hpDf761; daf-2(jlu2)*. Although *hpDf761*; *daf-2(jlu2); daf-28(tm2308)* animals constitutively formed dauers at all temperatures as expected [32], a small proportion (5–10%) spontaneously recovered on *E. coli* OP50. We therefore imaged these escapers as day 1 adults with synchronous controls. DAF-2B::mScarlet levels were elevated in *daf-2(jlu2); daf-28(tm2308)* adult animals and increased further with additional loss of ILP agonists (Fig 4C), indicating that loss of ILP agonists leads to increased nervous system DAF-2B in animals with functional UNC-31. In contrast, *ins-18* deletion significantly reduced nervous system DAF-2B::mScarlet levels in adult *unc-31(e928)* animals but did not affect wild type animals (Fig 4D). Thus, loss of an ILP antagonist (INS-18) or increased expression of an ILP agonist (DAF-28) reduces nervous system DAF-2B::mScarlet in the *unc-31* mutant background, indicating that increased DAF-2B levels are associated with reduced agonist ILP availability. The dauer formation and longevity phenotypes of *unc-31* mutants are suppressed by mutations in the FOXO transcription factor DAF-16 [21]. Correspondingly, we found that *daf-16* RNAi reduced nervous system DAF-2B::mScarlet levels in *unc-31* mutants (Fig 4E), as well as in the *hpDf761; daf-2(jlu2); daf-28(tm2308)* background (Fig 4F). Taken together, these data indicate that reduced IIS is a driver of increased nervous system DAF-2B::mScarlet, arising primarily from reduced levels of ILP agonists.

### DAF-2B heterodimers are detected in neurons

Our previous work suggests a model in which soluble DAF-2B homodimers are secreted into the extracellular environment where they sequester ILPs and limit their activity at the full-length DAF-2 receptor [5,16,33]. Subsequently, DAF-2B is cleared from the pseudocoelomic space by internalization into coelomocytes [5]. However, cell culture transfection experiments also demonstrated that DAF-2B monomers could form heterodimers with DAF-2A monomers [5]. These hybrid dimers would be expected to be expressed on the cell surface due to the transmembrane domain in DAF-2A. We therefore hypothesized that the nervous system DAF-2B::mScarlet signal could represent membrane-associated heterodimers between DAF-2B and full-length DAF-2 receptors.

**Fig 5:**
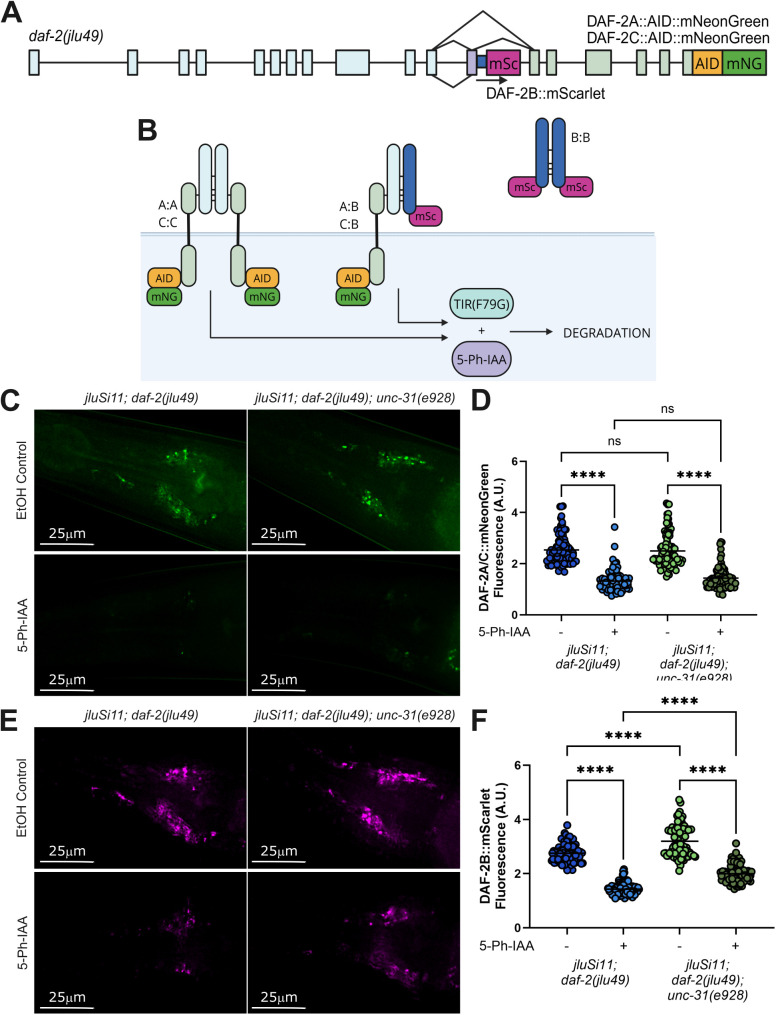
DAF-2B heterodimers are detected in neurons. **(A)** Alternative splicing produces *daf-2a/c::AID::mNeonGreen* and *daf-2b::mScarlet* transcripts from the *daf-2* genomic locus in *daf-2(jlu49).* **(B)** DAF-2 AA and DAF-2 AB dimers are degraded in the presence of the auxin analog 5-Ph-IAA. Degradation of AB heterodimers will also reduce DAF-2B::mScarlet but BB dimers will not be degraded. **(C)** Representative images of *jluSi11; daf-2(jlu49)* and *jluSi11; daf-2(jlu49); unc-31(e928)* day 1 adults illustrating the reduction in neuronal DAF-2A/C::AID::mNeonGreen after exposure to 10 µM 5-Ph-IAA for 5 h. **(D)** DAF-2A/C::AID::mNeonGreen is reduced in *jluSi11; daf-2(jlu49)* and *jluSi11; daf-2(jlu49); unc-31(e928)* animals treated with 10 µM 5-Ph-IAA. **(E)** Representative images of *jluSi11; daf-2(jlu49)* and *jluSi11; daf-2(jlu49); unc-31(e928)* day 1 adults illustrating the reduction in neuronal DAF-2B::mScarlet after exposure to 10 µM 5-Ph-IAA for 5 h. **(F)** DAF-2B::mScarlet is reduced in *jluSi11; daf-2(jlu49)* and *jluSi11; daf-2(jlu49); unc-31(e928)* animals treated with 10 µM 5-Ph-IAA. DAF-2B::mScarlet is higher in *jluSi11; daf-2(jlu49); unc-31(e928)* animals compared *with jluSi11; daf-2(jlu49)* both before and after auxin. For D & F, data are pooled from 4 biological replicates. One-way ANOVA with Sidak’s post-hoc test for indicated pair-wise comparisons, ns = not significant, **** p < 0.0001. Panels A and B were created in BioRender. Gill, M. (2026) https://BioRender.com/5hynawv.

Dimerization of DAF-2 is predicted to occur through the formation of inter-chain disulfide bonds between the ligand binding domains (α subunits) of receptor monomers during processing in the ER. To establish the existence of heterodimers containing truncated DAF-2B and full-length DAF-2 *in vivo*, we inserted mScarlet into the *daf-2b* genomic locus in *daf-2(hq363)*, a background in which the auxin-induced degron (AID) and mNeonGreen fluorescent protein had been inserted after exon 17 of the *daf-2* genomic locus [27] (Fig 5A). The resulting strain, *daf-2(jlu49),* which produces both DAF-2B::mScarlet and DAF-2A/C::AID::mNeonGreen from the same locus, was then crossed into jluSi11(rgef-1p::TIR1(F79G)::F2A::mTagBFP2::AID*::NLS::tbb-2 3’UTR)] to facilitate auxin induced degradation of DAF-2A/C in neurons. Since DAF-2B lacks a transmembrane domain, DAF-2B::mScarlet homodimers would be soluble in the ER lumen and undergo secretion to the extracellular environment (Fig 5B). On the other hand, if a DAF-2B monomer forms a heterodimer with a full-length DAF-2A or DAF-2C monomer, auxin-induced degradation of DAF-2A/C::AID::mNeonGreen would not only reduce the mNeonGreen fluorescent signal but would also result in a reduction of DAF-2B::mScarlet (Fig 5B).

To examine the effect of auxin treatment in the nervous system, we exposed day 1 adult *jluSi11; daf-2(jlu49)* and *jluSi11; daf-2(jlu49)*; *unc-31(e928)* animals to 10 µM 5-Ph-IAA, an auxin analog, for 5 h to induce degradation of DAF-2A/C. In untreated animals, there was no difference in DAF-2A/C::mNeonGreen fluorescence between wild type and *unc-31(e928)* (Fig 5C and 5D), consistent with the data in Fig 3C. 5-Ph-IAA treatment resulted in a significant reduction in DAF-2A/C::mNeonGreen fluorescence and the residual fluorescence was not different between wild type and *unc-31(e928)* (Fig 5C and 5D). As expected, DAF-2B::mScarlet was significantly higher in *jluSi11; daf-2(jlu49); unc-31(e928)* compared with controls (Fig 5E and 5F). 5-Ph-IAA treatment resulted in a significant reduction in neuronal DAF-2B::mScarlet in both wild type and *unc-31* mutants (Fig 5E and 5F), indicating that some of the neuronal DAF-2B::mScarlet signal is derived from covalently linked DAF-2A/C – DAF-2B heterodimers. In addition, the residual DAF-2B::mScarlet fluorescence after 5-Ph-IAA treatment was significantly higher in *unc-31* animals relative to wild type (Fig 5E and 5F).

We also crossed *jluSi11; daf-2(jlu49)* into the multiple ILP agonist deletion background, *hpDf761; daf-28(tm2308),* and observed a reduction in neuronal DAF-2B::mScarlet upon degradation of DAF-2A/C::AID::mNeonGreen by 5-Ph-IAA treatment (S3 Fig), providing evidence for DAF-2A/C - DAF-2B heterodimers in another genetic background. The residual DAF-2B::mScarlet fluorescence after 5-Ph-IAA treatment was also higher in *jluSi11; hpDf761; daf-2(jlu49); daf-28(tm2308)* animals (S3 Fig).

### FLIM-FRET confirms the existence of DAF-2B homodimers and heterodimers

**Fig 6:**
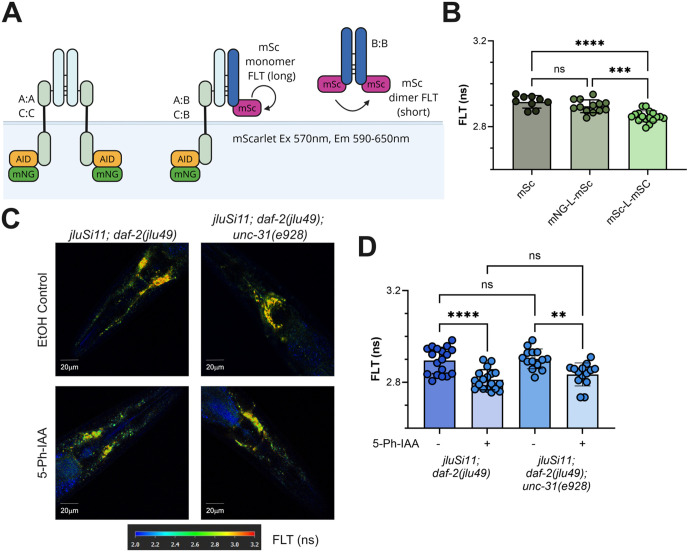
FLIM-FRET confirms the existence of DAF-2B homodimers and heterodimers. **(A)** DAF-2B::mScarlet in a heterodimer with DAF-2A/C will exhibit a fluorescence lifetime (FLT) similar to monomeric mScarlet (long), while DAF-2B::mScarlet in a BB homodimer will undergo FRET, thus reducing FLT. The diagram was created in BioRender. Gill, M. (2026) https://BioRender.com/5hynawv. **(B)** There is a significant reduction in mScarlet FLT in an mScarlet dimer expressed in neurons, compared with monomeric mScarlet and mScarlet in a tandem dimer with mNeonGreen. **(C)** Representative FLIM images of *jluSi11; daf-2(jlu49)* and *jluSi11; daf-2(jlu49); unc-31(e928)* after treatment with ethanol (EtOH) or 5-Ph-IAA illustrating DAF-2B::mScarlet FLT in the nervous system. **(D)** DAF-2B::mScarlet FLT in *jluSi11; daf-2(jlu49)* and *jluSi11; daf-2(jlu49); unc-31(e928)* is significantly reduced after treatment with 10 µM 5-Ph-IAA compared with ethanol control. There is no difference in DAF-2B::mScarlet FLT between *jluSi11; daf-2(jlu49)* and *jluSi11; daf-2(jlu49); unc-31(e928)* under ethanol control conditions or after 10 µM 5-Ph-IAA treatment. Data are pooled from 2 biological replicates. One-way ANOVA with Sidak’s post-hoc test for indicated pair-wise comparisons, ns = not significant, ** p < 0.01, *** p < 0.001, **** p < 0.0001.

As an orthogonal approach to confirm the existence of DAF-2B homodimers and heterodimers in the nervous system, we employed fluorescence lifetime imaging microscopy - Förster resonance energy transfer (FLIM-FRET) on a Leica Stellaris 8 FALCON confocal microscope [34] to monitor homoFRET of DAF-2B::mScarlet. Fluorescence lifetime (FLT) is an intrinsic photophysical property of a fluorophore and, unlike intensity-based measurements, is independent of expression level which makes FLIM a robust and quantitative approach for detecting protein-protein interactions *in vivo* [35–37]. In the case of DAF-2B::mScarlet, both donor and acceptor fluorophores are identical; therefore, energy transfer arising from dimerization is detected as a reduction in donor FLT (Fig 6A). Furthermore, the FLIM signal will be the ensemble average of all fluorophore signals within any given pixel, so changes in FLT can indicate corresponding changes in the relative distribution of long FLT (monomeric) and short FLT (dimeric) populations.

To establish *in vivo* benchmarks for mScarlet FLT and homoFRET, we used the neuronal *rab-3* promoter to express monomeric mScarlet (mSc *- rab-3p::*mScarlet::*rab-3* 3’UTR), dimeric mScarlet (mSc-L-mSc - *rab-3p::*mScarlet-30aa linker-mScarlet::*rab-3* 3’UTR) and a tandem dimer of mNeonGreen and mScarlet (mNG-L-mSc - *rab-3p::*mNeonGreen-30aa linker-mScarlet::*rab-3* 3’UTR, a direct control for DAF-2A/C::AID::mNG – DAF-2B::mSc heterodimer) in the nervous system. The FLT of monomeric mScarlet and mScarlet in the tandem dimer were not significantly different (mSc FLT = 2.916 ns; mNG-L-mSc = 2.896 ns) indicating that the presence of mNeonGreen in a dimer does not interfere with mScarlet FLT (Fig 6B). The FLT of dimeric mScarlet was significantly reduced (mSc-L-mSc = 2.848 ns) compared with the monomers (Fig 6B), consistent with homoFRET occurring in the dimer.

In *jluSi11; daf-2(jlu49)* and *jluSi11; daf-2(jlu49); unc-31(e928)* animals exposed to ethanol, the FLT of DAF-2B::mScarlet was 2.891 ns and 2.903 ns (Fig 6C and 6D), values that were not statistically different from the monomeric mScarlet controls (S4A Fig). This is consistent with DAF-2B::mScarlet predominantly within a heterodimer with DAF-2A/C (Fig 6A). In animals exposed to 5-Ph-IAA for 5 h to deplete DAF-2B::mScarlet found in heterodimers with full-length DAF-2A/C::AID::mNeonGreen, there was a significant reduction in mScarlet FLT compared to the ethanol control, in both wild type (Control = 2.891 ns vs 5-Ph-IAA = 2.811 ns) and the *unc-31* mutant (Control = 2.903 vs 5-Ph-IAA = 2.834 ns) (Fig 6C and 6D). The FLT values after auxin treatment were not significantly different from the mScarlet dimer FLT (S4A Fig). These data are consistent with the depletion of heterodimers by 5-Ph-IAA and an enrichment of the DAF-2B homodimer population in the residual mScarlet signal (Fig 6A). The depletion of DAF-2A/C::AID::mNeonGreen in these FLIM images was confirmed via integrated total photon counts (S4B Fig).

### DAF-2B undergoes neuronal endocytosis in *unc-31* mutants

**Fig 7:**
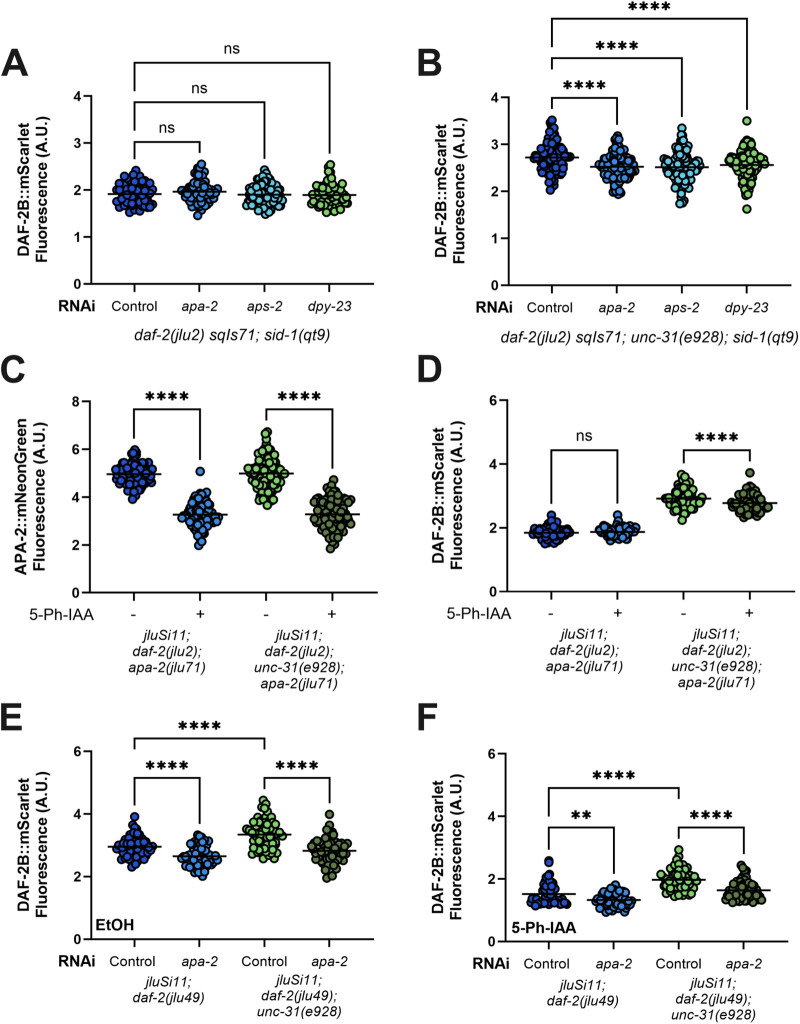
DAF-2B undergoes AP2-dependent endocytosis in *unc-31* mutants. **(A)** Neuron-specific RNAi of *apa-2, aps-2* and *dpy-23* has no effect on DAF-2B::mScarlet levels in *daf-2(jlu2) sqIs71; sid-1(qt9)* worms. **(B)** Neuron-specific RNAi of *apa-2, aps-2* and *dpy-23* reduces DAF-2B::mScarlet levels in *daf-2(jlu2) sqIs71; unc-31(e928); sid-1(qt9)* worms. **(C)** Neuronal APA-2::mNeonGreen::AID levels are reduced after treatment with 5-Ph-IAA for 5 h in both wild type and *unc-31* mutant backgrounds. **(D)** DAF-2B::mScarlet levels are reduced in *daf-2(jlu2); unc-31(e928); apa-2(jlu71)* mutants, but not in *daf-2(jlu2); apa-2(jlu71)* worms, after treatment with 5-Ph-IAA for 5 h. **(E)** Systemic *apa-2* RNAi reduces expression of neuronal DAF-2B::mScarlet in *jluSi11; daf-2(jlu49)* and *jluSi11; daf-2(jlu49); unc-31(e928)* day 1 adults treated with ethanol. **(F)** Systemic *apa-2* RNAi reduces expression of neuronal DAF-2B::mScarlet in *jluSi11; daf-2(jlu49)* and *jluSi11; daf-2(jlu49); unc-31(e928)* day 1 adults treated with 10 µM 5-Ph-IAA. Data are pooled from 3 (A, E, F), 4 (C,D) or 5 (B) biological replicates. One-way ANOVA with Sidak’s post-hoc test for indicated pair-wise comparisons, ns = not significant, * p < 0.05, ** p < 0.01, **** p < 0.0001.

DAF-2B homodimers lack a transmembrane domain and thus are expected to be secreted into the extracellular environment where they undergo endocytosis in coelomocytes. We therefore considered the possibility that neurons may also play a role in clearing DAF-2B via endocytosis and that elevated nervous system DAF-2B::mScarlet in *unc-31* animals corresponds to internalized DAF-2B. There are several mechanisms by which extracellular material is internalized, with clathrin-mediated endocytosis being common [38]. This process involves the binding of extracellular cargo to adapter proteins of the AP2 complex. We therefore examined the effect of RNAi of AP2 adaptor complex subunits *(apa-2, aps-2,* and *dpy-23*) in a neuron-specific RNAi background [39]. In wild type worms, nervous system DAF-2B::mScarlet was unaffected by RNAi, but in the *unc-31* background neuronal DAF-2B::mScarlet was significantly reduced by RNAi of *apa-2, aps-2* and *dpy-23* (Fig 7A and 7B).

To confirm AP2-dependent endocytosis of DAF-2B::mScarlet, we also examined the effect of neuronal degradation of APA-2, by generating a strain that expresses mNeonGreen and the AID epitope at the C-terminus of endogenous APA-2 protein. APA-2::mNeonGreen::AID fluorescence was detected in the nervous system (S5A Fig) as previously reported [40] and we observed a significant reduction in mNeonGreen fluorescence after treatment with 5-Ph-IAA for 5 h (Fig 7C). Treatment with 5-Ph-IAA did not affect DAF-2B::mScarlet levels in the wild type *daf-2(jlu2)* background, but there was a significant reduction in DAF-2B::mScarlet in *unc-31* animals following APA-2::mNeonGreen::AID degradation (Fig 7D). Similar results were obtained with exposure to 5-Ph-IAA for 24 h from L4 (Fig S5B–S5D), confirming that DAF-2B undergoes AP2-dependent neuronal endocytosis in the *unc-31* background.

We then wanted to determine the effect of *apa-2* RNAi after depletion of DAF-2B heterodimers with 5-Ph-IAA treatment. We were unable to perform this experiment in the neuron-specific RNAi background due to a genetic interaction between *sqIs71; sid-1(qt9)* in the *jluSi11; daf-2(jlu49)* and *jluSi11; daf-2(jlu49)*; *unc-31(e928)* backgrounds that resulted in a Daf-c phenotype. However, we determined that systemic *apa-2* RNAi was effective in reducing APA-2::mNeonGreen::AID in the nervous system (S5E Fig), so we used this approach in *jluSi11; daf-2(jlu49)* and *jluSi11; daf-2(jlu49)*; *unc-31(e928)* animals. In ethanol-treated vehicle controls, systemic *apa-2* RNAi resulted in a significant reduction in DAF-2B::mScarlet in both wild type and *unc-31* mutant backgrounds (Fig 7E). After depletion of heterodimers by 5-Ph-IAA treatment, the remaining DAF-2B::mScarlet signal was significantly higher in *unc-31* mutants compared to wild type (Fig 7F), as previously observed (Fig 5F). *apa-2* RNAi was associated with a further decrease in the remaining DAF-2B::mScarlet in both backgrounds (Fig 7F). We also observed an *apa-2-*dependent reduction in residual DAF-2B::mScarlet in the *jluSi11; hpDf761; daf-2(jlu49); daf-28(tm2308)* background following auxin treatment (S6 Fig).

**Fig 8:**
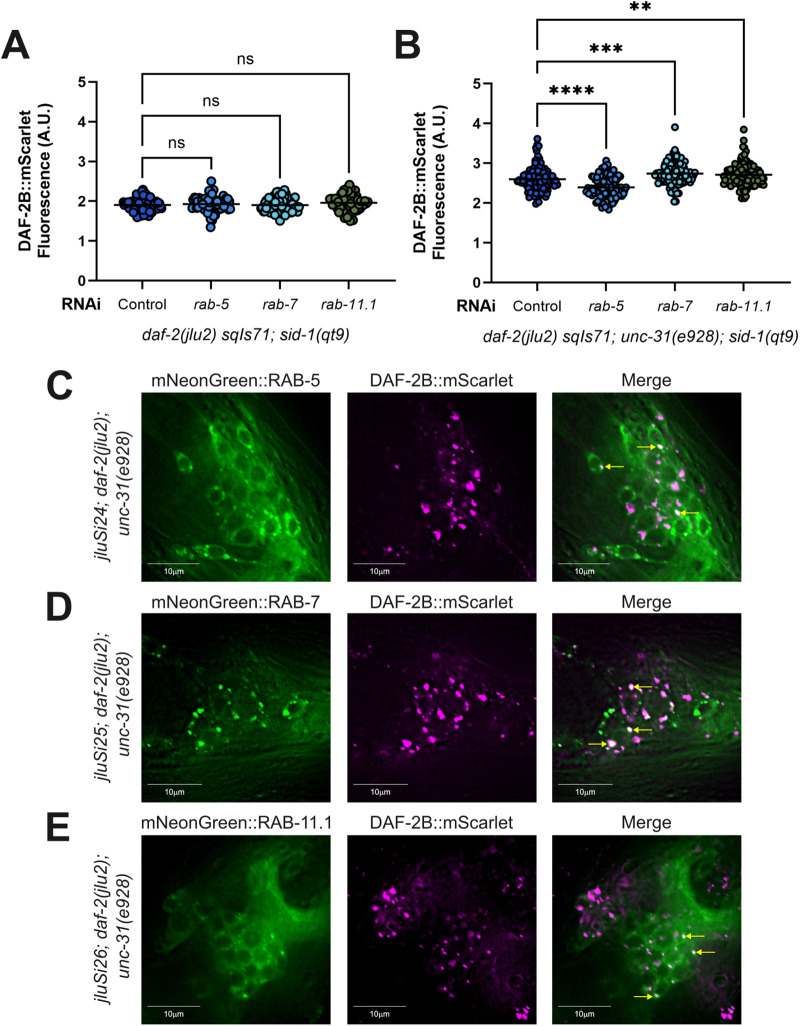
DAF-2B endocytosis in *unc-31* mutants involves neuronal *rab-5*, *rab-7* and *rab-11.1.* **(A)** Neuron-specific RNAi of *rab-5, rab-7* or *rab-11.1* has no effect on DAF-2B::mScarlet levels in *daf-2(jlu2) sqIs71; sid-1(qt9)* worms. **(B**) Neuron-specific RNAi of *rab-5* reduces DAF-2B::mScarlet levels, while *rab-7* and *rab-11.1* increases DAF-2B::mScarlet levels in *daf-2(jlu2) sqIs71; unc-31(e928); sid-1(qt9)* worms. DAF-2B::mScarlet co-localizes (indicated by yellow arrows) with neuronally expressed mNeonGreen::RAB-5 **(C)**, mNeonGreen::RAB-7 **(D)** and mNeonGreen::RAB-11.1 **(E)**. Data are pooled from 3 (A) or 5 (B) biological replicates. One-way ANOVA with Sidak’s post-hoc test for indicated pair-wise comparisons, ns = not significant, ** p < 0.01, *** p < 0.001, **** p < 0.0001. Images in C-E are representative of n = 30 images from 3 biological replicates for each reporter.

To further examine endosomal trafficking of DAF-2B, we performed RNAi against *rab-5* (early endosome), *rab-7* (late endosome), as well as *rab-11.1* (recycling endosome) [38]. Using the *sqIs71; sid-1(qt9)* neuron-specific RNAi background, we did not observe any effect of *rab* RNAi in wild type *daf-2(jlu2)* animals (Fig 8A). However, in the *daf-2(jlu2); unc-31(e928)* background we found that *rab-5* RNAi reduced DAF-2B:mScarlet expression, while *rab-7* and *rab-11.1* RNAi resulted in increased DAF-2B::mScarlet (Fig 8B). To confirm that DAF-2B::mScarlet localizes to the neuronal endosomal pathway, we generated N-terminal mNeonGreen-tagged RAB-5, RAB-7, and RAB-11.1 as single copy integrations driven by the neuron-specific *rab-3p* promoter. We observed neuronal colocalization of DAF-2B::mScarlet with mNeonGreen::RAB-5, mNeonGreen::RAB-7, and mNeonGreen::RAB-11.1 in the *unc-31* background (Fig 8C–8E), confirming that DAF-2B is trafficked through the endosomal pathway of neurons.

### Functional loss of DAF-2B by ER retention promotes IIS

**Fig 9:**
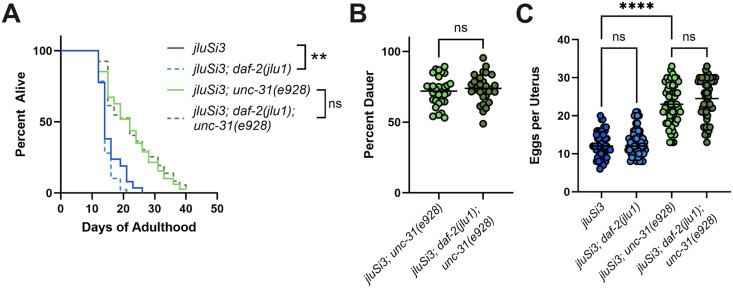
*daf-2b* deletion has no effect on *unc-31* phenotypes. **(A)** *daf-2b* deletion in *jluSi3; daf-2(jlu1)* does not affect lifespan in *unc-31(e928)* mutants. Data are representative of 3 biological replicates. Log rank test, ns = not significant, ** p < 0.01. **(B)** *daf-2b* deletion does not affect dauer entry at 26.1°C in *unc-31(e928)* mutants. Data are pooled from 3 biological replicates. Student’s *t*-test ns = not significant. **(C)** *daf-2b* deletion does not affect egg retention in *unc-31(e928)* mutants. Data are pooled from 3 biological replicates (B,C). One-way ANOVA with Sidak’s post-hoc test for indicates pair-wise comparisons, ns = not significant, **** p < 0.0001.

We have previously shown that overexpression of DAF-2B increased longevity in wild type worms and increased dauer formation in *daf-2(e1368)* mutants that have reduced IIS [5]. To examine the contribution of elevated DAF-2B to *unc-31* mutant phenotypes, we crossed *unc-31(e928)* with a *daf-2b* deletion strain, *jluSi3; daf-2(jlu1)*, in which *daf-2(jlu1)* lacks both *daf-2c* and *daf-2b* due to deletion of the genomic sequence between exon 11 and exon 12, and *jluSi3* restores *daf-2c* expression by single copy insertion of *daf-2c* cDNA from a 3kb *daf-2* promoter at a Mos insertion site [5] (S7 Fig). Comparing *jluSi3; daf-2(jlu1); unc-31(e928)* animals with *jluSi3; unc-31(e928)* controls, we found no difference in lifespan in 2 out of 3 replicate experiments (Figs 9A and S8) or in dauer formation (Fig 9B). *unc-31* mutants also exhibit an egg-laying defective (Egl) phenotype which manifests as increased retention of eggs in the uterus, and there was also no effect of *jluSi3; daf-2(jlu1)* on egg retention in the *unc-31(e928)* mutant background (Fig 9C).

**Fig 10:**
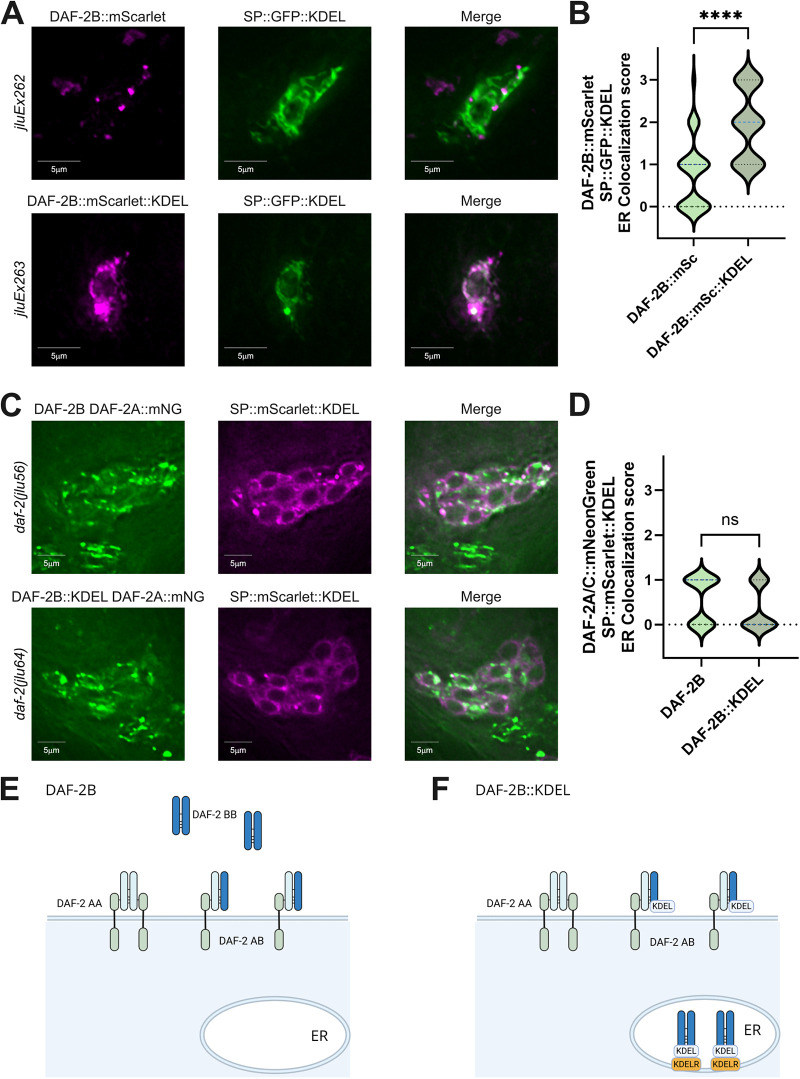
DAF-2A trafficking is not affected by DAF-2B::KDEL. **(A)** DAF-2B::mScarlet in the nervous system, expressed from the *jluEx262[daf-2p::DAF-2B::mScarlet]* array, does not colocalize with an SP::GFP::KDEL ER marker expressed from the *daf-2* promoter. However, DAF-2B::mScarlet::KDEL in the nervous system expressed from the *jluEx263[daf-2p::DAF-2B::mScarlet::KDEL]* array shows colocalization with an SP::GFP::KDEL marker indicating retention in the ER. **(B)** Colocalization scores are significantly higher in in animals expressing DAF-2B::mScarlet::KDEL. **(C)** In a wild type DAF-2B background, *daf-2(jlu56),* DAF-2A/C::mNeonGreen does not colocalize with an SP::mScarlet::KDEL ER marker in the nervous system. Similarly, in a DAF-2B::KDEL mutant background, *daf-2(jlu64),* DAF-2A/C::mNeonGreen does not colocalize with an SP::mScarlet::KDEL ER marker in the nervous system. **(D)** Colocalization scores between DAF-2A/C::AID::mNeonGreen and the SP::mScarlet::KDEL ER marker are not affected by DAF-2B::KDEL. **(E)** In wild type worms, DAF-2B exists as a secreted homodimer and a membrane-bound heterodimer. **(F)** In the *daf-2(jlu44)* mutant, DAF-2B homodimers are retained in the ER, while trafficking of DAF-2B / DAF-2A heterodimers is unaffected. For B & D, images were manually scored for extent of co-localization and assigned a score (0 – none, 1 – low, 2 – moderate, 3 – high). Data are pooled from 3 biological replicates. Student’s t test, **** p < 0.0001. Panels E and F were created in BioRender. Gill, M. (2026) https://BioRender.com/5hynawv.

In an alternate strategy to render animals deficient in DAF-2B, we used CRISPR-Cas9 to add a KDEL sequence to the C-terminus of DAF-2B, generating *daf-2(jlu44[daf-2b::kdel])* (S7 Fig). The KDEL motif is found on soluble ER resident proteins and interacts with the KDEL receptor to facilitate ER retention [41,42]. We reasoned that DAF-2B::KDEL would be retained in the ER, therefore preventing DAF-2B secretion into the extracellular space. To verify ER-retention of DAF-2B::KDEL, we generated extrachromosomal array lines and found increased colocalization of *daf-2p*::DAF-2B::mScarlet::KDEL with a GFP ER marker compared with wild type *daf-2p*::DAF-2B::mScarlet arrays (Fig 10A and 10B). To examine the effect of DAF-2B::KDEL on trafficking of heterodimer complexes, we examined *daf-2a/c::AID::mNeonGreen* expression in animals expressing a polycistronic mScarlet ER marker (SL2::SP::mScarlet::KDEL). We did not observe any difference in co-localization of DAF-2A/C::AID::mNeonGreen and the mScarlet ER marker between *daf-2(jlu56)* animals with wild type DAF-2B and *daf-2(jlu64)* animals expressing DAF-2B::KDEL (Fig 10C and 10D). These data suggest that DAF-2B::KDEL promotes ER retention of DAF-2B homodimers without affecting the trafficking of heterodimers (Fig 10E and 10F). The addition of the KDEL sequence to DAF-2B in *daf-2(jlu44)* mutants did not change expression of *hsp-4* transcripts (S9 Fig), indicating that there is no induction of the ER stress response.

**Fig 11:**
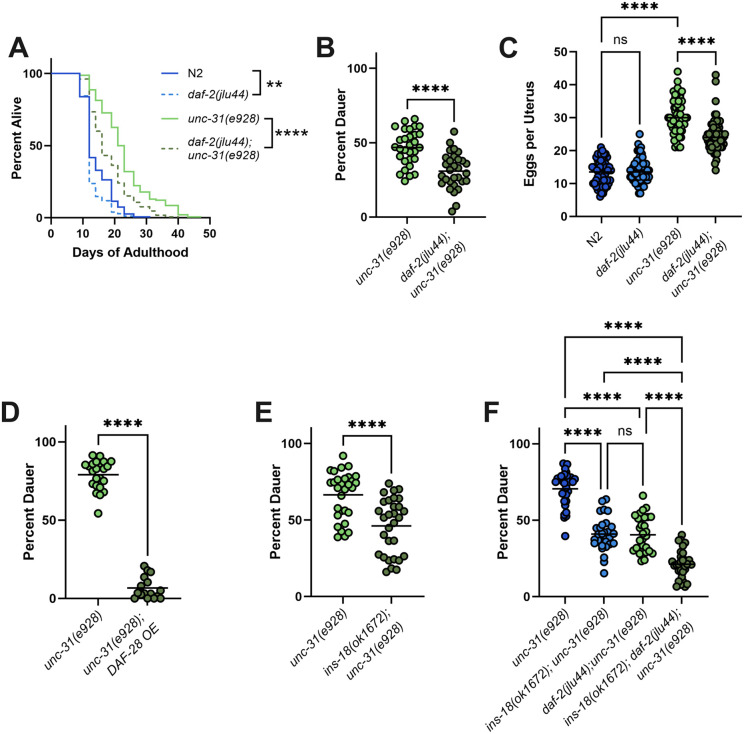
Functional loss of DAF-2B by ER retention promotes IIS. **(A)** ER retention of DAF-2B::KDEL reduces lifespan in N2 and *unc-31(e928).* Data are representative of 3 biological replicates. Log rank test, ** p < 0.01, **** p < 0.0001. **(B)** ER retention of DAF-2B::KDEL in *daf-2(jlu44)* reduces dauer formation in *unc-31(e928).* Data are pooled from 3 biological replicates. Student’s *t*-test **** p < 0.0001. **(C)** ER retention of DAF-2B::KDEL in *daf-2(jlu44)* reduces egg retention in *unc-31(e928)* mutants. Data are pooled from 3 biological replicates. One-way ANOVA with Sidak’s post-hoc test for indicated pair-wise comparisons, ns = not significant, **** p < 0.0001. **(D**) Overexpression of DAF-28 suppresses dauer formation in *unc-31(e928)* mutants at 26.1°C. Data are pooled from 2 biological replicates. Student’s *t*-test*,* **** p < 0.0001*.* **(E)** Deletion of *ins-18* suppresses dauer entry in *unc-31(e928)* mutants at 26.1°C. Data are pooled from 3 biological replicates*.* Student’s *t*-test*,* **** p < 0.0001*.* **(F)** *daf-2(jlu44)* and deletion of *ins-18* are additive in suppressing dauer forma*t*ion in *unc-31(e928)* mutants at 26.1°C. Data are pooled from 3 biological replicates*.* One-way ANOVA with Sidak’s post-hoc test for pair-wise comparisons, ns = not significant, **** p < 0.0001.

We found that *daf-2(jlu44)* significantly reduced lifespan in 2 of 3 replicate experiments in wild type and 3 of 3 replicate experiments in *unc-31(e928)* mutants (Figs 11A and S8), as well as reducing dauer formation in *unc-31* mutants (Fig 11B). We also found that *daf-2(jlu44)* reduced egg retention in *unc-31* mutants, as did *daf-16* RNAi (Figs 11C and S10). In Fig 4, we found that DAF-2B::mScarlet levels in *unc-31* mutants were reduced by both DAF-28 overexpression and deletion of *ins-18*. Correspondingly, we found that dauer formation in *unc-31* mutants can be suppressed by either DAF-28 overexpression or *ins-18* deletion (Fig 11D and 11E), indicating that increased availability of ILP agonists can rescue dauer formation in *unc-31* mutants. To determine whether DAF-2B::KDEL leads to increased availability of agonist ILPs, we examined dauer formation in *ins-18(ok1672); daf-2(jlu44); unc-31(e928)* mutants. Dauer formation was suppressed to the same extent in *ins-18(ok1672); unc-31(e928)* and *daf-2(jlu44); unc-31(e928)* double mutants but was further suppressed in the *ins-18(ok1672); daf-2(jlu44); unc-31(e928)* triple mutant background (Fig 11F). This suggests the *daf-2(jlu44)* mutation promotes IIS by increasing agonist ILP availability.

## Discussion

### Identification of a mutant that increases DAF-2B

In this study, we used forward mutagenesis to identify regulators of DAF-2B expression in an unbiased manner. We isolated a mutant with increased DAF-2B::mScarlet signal in the nervous system of adult animals and subsequent phenotypic characterization, genetic complementation and sequencing determined that the causal mutation was in the *unc-31* gene. *unc-31* mutants have been characterized extensively and have defects in dense core vesicle exocytosis and secretion of neuropeptides and monoamines [17–20,43]. The 13kb *unc-31a* genomic sequence comprises 20 exons and the 4kb mRNA encodes a 1453 amino acid protein [18] containing multiple domains that facilitate interaction with the plasma membrane (C2 & PH domains), interaction with SNARE proteins (Munc Homology Domain, MHD) and binding to DCV (DCVBD) [17–19]. The mutation identified in this study is a premature stop codon in the MHD and therefore is predicted to generate a protein that also lacks the DCVBD (S1 Fig). Given that the well-characterized null allele, *e928*, contains a large deletion that spans the MHD and DCVBD [18], it is likely that our allele, *jlu22*, is also a null mutation.

### Reduced IIS leads to increased nervous system DAF-2B

Secreted proteins are trafficked through the ER and Golgi and subsequently enter the constitutive secretory pathway or the regulated secretory pathway [38], the latter involving DCVs and UNC-31. The first 11 exons of *daf-2b* mRNA are identical to full length *daf-2* transcripts, and therefore the *daf-2b* transcript contains a signal peptide sequence and is expected to be processed in the ER and trafficked to the cell surface via the constitutive secretory pathway, like full-length receptors. Since this trafficking pathway does not involve DCVs or UNC-31, we did not expect increased nervous system DAF-2B to be a result of a secretion defect in *unc-31* mutants. Two observations further support this. First, using the well-validated model of fluorescent protein accumulation in coelomocytes as a marker of secretion, we found that DAF-2B secretion was elevated in *unc-31* mutants, rather than reduced (Fig 3). Second, genetic loss of ILP agonists in animals otherwise wild type for UNC-31 also increased DAF-2B::mScarlet levels in the nervous system (Fig 4).

The defect in DCV docking in *unc-31* mutants broadly impacts many neuropeptides including ILPs. However, *unc-31* mutants do not appear to be entirely deficient in ILP secretion. Phenotypes of *unc-31* mutants are relatively weak compared to more severe IIS defects [32,44] and the dauer and lifespan phenotypes are generally consistent with reduced agonist secretion [12,32]. In addition, there is evidence for UNC-31-independent mechanisms for ILP secretion. For instance, single cell transcript analysis indicates that many ILPs are expressed in neurons that don’t express UNC-31 [8], and other factors involved in ILP secretion have been identified, including ASNA-1, which confers a more profound arrest phenotype when mutated [29]. It is also possible that UNC-31 is only involved in secretion of a subset of ILPs. In this respect, reduced secretion of the ILP agonist DAF-28 has been shown in *unc-31* mutants [22], but a recent report suggests that DAF-28 secretion is UNC-31-independent [45]. In this study, we find that both DAF-28 overexpression and deletion of the ILP antagonist *ins-18* can suppress the dauer phenotype of *unc-31,* implying that the ability to secrete at least some DAF-28 and INS-18 is maintained in these mutants. Deletion of *ins-18* and overexpression of DAF-28 reduced nervous system DAF-2B::mScarlet levels in *unc-31* mutants (Fig 4), while loss of multiple ILP agonists, in an otherwise wild type background, was sufficient to increase DAF-2B::mScarlet levels (Fig 4). These data suggest that reduced IIS arising from reduced agonist ILP secretion is the main driver of increased DAF-2B in the nervous system. This was further supported by the observation that *daf-16* RNAi in *unc-31* mutants, as well as in the multiple ILP agonist deletion background, suppressed neuronal DAF-2B expression (Fig 4E and 4F). Taken together, these data suggest that elevated DAF-2B plays a role in environments that reduce IIS, by limiting the activity of ILPs.

### DAF-2B heterodimers are detected in the nervous system

The ligand binding domain (α subunit) of DAF-2A, DAF-2C and DAF-2B are identical, except for short C-terminal extensions in DAF-2B and DAF-2C. Importantly, the conserved cysteine residues involved in disulfide bond formation during dimerization are present in all three isoforms. Using transfection studies in mammalian cell culture, we have previously shown that DAF-2A and DAF-2B can form covalently linked heterodimers [5]. Thus, we considered the possibility that some fraction of the nervous system DAF-2B signal represented heterodimers between DAF-2B and full-length DAF-2 receptors at the cell membrane. In this respect, the loss of DAF-2B::mScarlet signal upon AID of full-length DAF-2A/C::AID::mNeonGreen is strongly suggestive of the presence of heterodimers. Interestingly, the amount of DAF-2B::mScarlet remaining after depletion of heterodimers was higher in both *unc-31* mutants and the multi-ILP agonist deletion background. This may suggest that DAF-2B homodimers are elevated in mutants with reduced insulin signaling. However, we cannot rule out the possibility that this fraction also contains heterodimers that escape degradation. Since TIR1 protein expression is driven by the *rgef-1* promoter, and *daf-2* is expressed in some neurons that lack *rgef-1* [8]*,* it is possible that there will be cells in the nervous system that escape auxin-induced DAF-2 degradation.

As an orthogonal approach to confirm the existence of heterodimers we used FLIM-FRET, which provides a sensitive and quantitative approach to probe receptor assembly *in vivo*. Our FLIM-FRET analysis revealed that the FLT of DAF-2B::mScarlet under basal conditions is consistent with the presence of a monomeric mScarlet species, supporting the existence of hybrid DAF-2A/C - DAF-2B receptor dimers. Upon auxin treatment, which depletes DAF-2A/C::mNeonGreen and disrupts DAF-2A/C – DAF-2B heterodimers, we observed a reduction in FLT, consistent with an enrichment of DAF-2B homodimers which constrain mScarlet fluorophores into closer proximity, thereby enhancing energy transfer efficiency. These results highlight that FLIM-FRET is particularly powerful for detecting shifts in the ensemble distribution of monomeric versus dimeric states. *C. elegans* exhibits strong intrinsic autofluorescence from gut granules and other tissues, which poses a significant challenge for FLIM due to its short FLT. To address this, we applied two-component FLT fits to distinguish autofluorescence from the DAF-2B::mScarlet signal within the defined regions of interest (ROIs). Taken together, these data demonstrate that FLIM-FRET can resolve changes in the oligomeric state of an endogenously expressed receptor isoform *in vivo*, providing mechanistic insight into isoform-dependent assembly of the DAF-2 receptor.

Prior FLIM‑FRET studies have quantified endogenous protein-protein interactions either at endogenous expression levels in intact organisms [46,47] or in human tissues using antibody‑based FLIM‑FRET [48], and multiple animal reporter lines validate FLT-based FRET readouts *in vivo* [49,50]. Nevertheless, we were unable to identify reports resolving isoform‑specific receptor oligomerization at endogenous levels in a live animal by FLIM‑FRET [51], underscoring the utility of our DAF‑2B::mScarlet FLT shifts to infer isoform‑dependent assembly *in vivo*.

In mammals, there are two insulin receptor isoforms, IR-A and IR-B, which differ by the inclusion of an alternatively spliced cassette exon in IR-B that leads to a short C-terminal extension at the end of the ligand binding domain [52], analogous to the difference between DAF-2A and DAF-2C [6]. The functional consequence of this difference is that IR-A has a higher affinity for IGF-II than IR-B [52]. In mammals, there is also considerable evidence for the existence of heterodimers between IR and the closely-related IGF-1 receptor [53]. Since DAF-2A, DAF-2C and DAF-2B differ in the sequence of the α-subunit C-terminus, it is possible that each homodimeric receptor complex has different binding affinity for ILPs. By extension, a DAF-2A/C - DAF-2B heterodimer may have altered binding affinity for ILPs compared with the other homodimeric species. *C. elegans* has an unusually large set of ILPs that have diverse sequences and predicted structures [2,3]. Thus, the presence of different DAF-2 α subunit sequences and the ability to form both homodimers and heterodimers would provide a means of increasing selectivity and specificity of ILP binding. In humans, the IR is activated by trans-autophosphorylation following ligand binding and heterozygous mutations in the tyrosine kinase domain act in a dominant negative manner to inhibit insulin signaling [54,55]. Based on this evidence, we expect DAF-2B heterodimers to be non-signaling and therefore act as a new class of decoy receptor that functions to sequester ILPs.

### DAF-2B is subject to neuronal endocytosis

In *C. elegans*, secreted proteins are removed from the pseudocoelomic space by endocytosis in the coelomocytes, including ILPs and DAF-2B [5,28,29]. Here, we show DAF-2B::mScarlet also undergoes endocytosis in neurons. We found that loss of the AP2 adaptor protein complex subunits by neuron-specific RNAi, as well as by neuron-specific AID of APA-2, in an *unc-31* background decreased neuronal DAF-2B accumulation. Endocytic cargo has multiple fates within the cell, determined by the expression of small Rab GTPases on the surface of intracellular vesicles [38]. To this end, we found that neuronal loss of *rab-7* and *rab-11.1* resulted in elevated DAF-2B::mScarlet, whereas knockdown of *rab-5* decreased DAF-2B::mScarlet. We were also able to track DAF-2B::mScarlet through the endocytic pathway in *unc-31* mutants by colocalization with neuronally expressed Rab proteins tagged with mNeonGreen. In addition to being a marker of the early endosome, RAB-5 has been shown to be a positive regulator of the early stages of clathrin mediated endocytosis [56], which could explain the reduction in DAF-2B::mScarlet upon *rab-5* RNAi. The effects of *rab-7 and rab-11.1* RNAi suggest that DAF-2B::mScarlet is trafficked through both the late endosome / lysosome pathway (RAB-7) for degradation, with some fraction also entering the recycling endosome pathway (RAB-11.1) [38].

Endocytosis of membrane-bound insulin / IGF receptors is a well-established mechanism by which insulin signaling can be regulated [57]. In this context, adaptor proteins recognize and recruit activated receptors to endocytic vesicles in a clathrin-dependent manner [57]. Our experiments with DAF-2B::mScarlet cannot explicitly distinguish between endocytosis of heterodimers or homodimers. However, if heterodimers undergo AP2 dependent endocytosis, then we might expect to see no change or even an increase in DAF-2B::mScarlet upon loss of APA-2, since DAF-2B::mScarlet would remain at the membrane in a complex with DAF-2A. Precedence for this comes from a study that identified CHN-1, a ubiquitin ligase that is involved in internalization of activated DAF-2 receptors [58]. *chn-1* mutants have increased levels of DAF-2 and exhibit reduced lifespan consistent with a gain of function effect on insulin signaling. The mechanism by which soluble DAF-2B homodimers may be internalized in an AP2-dependent manner is not clear, but there may be additional protein binding partners that facilitate interaction with the AP2 complex.

We propose that one function of DAF-2B endocytosis is to clear ILPs from the extracellular space and thereby modify insulin signaling. As the endosome acidifies, the DAF-2B / ILP complex likely dissociates and a fraction of DAF-2B could be recycled back to the cell surface through a RAB-11.1 dependent process. Another fraction of DAF-2B is trafficked through RAB-7 coated vesicles, presumably for degradation, whether ILPs are bound or not. Since mScarlet fluorescence is sensitive to pH, we cannot differentiate between changes in DAF-2B::mScarlet fluorescence that arise from a change in subcellular compartmentalization or protein levels themselves. RAB-7 late endosomes are more acidic than other endosomal vesicles and therefore we may be underestimating the amount of DAF-2B::mScarlet in this compartment. Similarly, the reduction in DAF-2B::mScarlet fluorescence we observed in *unc-31* mutants overexpressing DAF-28 (Fig 4A) could be explained by an increase in DAF-2B endocytosis through the late endosome pathway and a corresponding quenching of mScarlet fluorescence in the acidic environment, rather than changes in protein levels.

### DAF-2B homodimers function to sequester ILP agonists and reinforce reduced IIS

We found different effects of the *daf-2b* mutants, *jluSi3; daf-2(jlu1)* and *daf-2(jlu44),* on *unc-31* phenotypes. Global deletion of *daf-2b* using *jluSi3; daf-2(jlu1)* in the *unc-31* mutant background has no effect on IIS, whereas the addition of an ER retention epitope to generate DAF-2B::KDEL in the *daf-2(jlu44)* mutant leads to an increase in IIS. Colocalization studies indicate that the KDEL sequence differentially affects DAF-2B homodimers and heterodimers, such that DAF-2B::KDEL homodimers are effectively retained in the ER, while DAF-2A/C - DAF-2B::KDEL heterodimers escape ER retention, presumably due to a failure of the KDEL receptor to bind DAF-2B::KDEL when it is held close to the membrane in a complex with DAF-2A. This would lead to a functional deficiency of DAF-2B homodimers while maintaining DAF-2B heterodimer expression. Importantly, the KDEL mutant did not show any change in *hsp-4* transcripts, indicating that the ER unfolded protein response was not induced due to an increased load of ER protein (S9 Fig).

**Fig 12:**
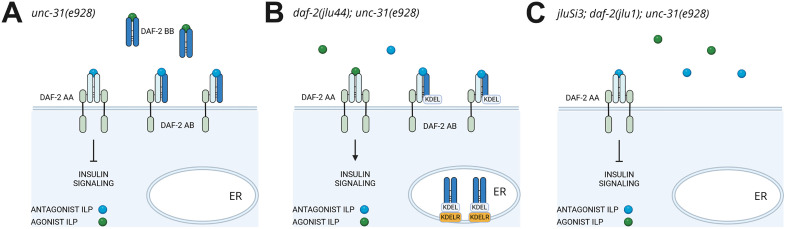
Model for the effect of DAF-2B homodimers and heterodimers on IIS in *unc-31* mutants. **(A)** Reduced insulin signaling in *unc-31(e928)* mutants is a consequence of reduced agonist ILP secretion and a relative increase in antagonist ILPs and leads to increased neuronal DAF-2B. DAF-2B is partitioned between secreted BB homodimers and cell-surface AB heterodimers. DAF-2B homodimers are elevated in *unc-31* mutants compared to wild type and function to sequester agonists ILPs, helping to reinforce reduced IIS. **(B)** We hypothesize that DAF-2A - DAF-2B::KDEL heterodimers escape ER retention while DAF-2B::KDEL homodimers are effectively retained in the ER. If DAF-2B homodimers preferentially bind ILP agonists and heterodimers sequester antagonists, functional loss of DAF-2B homodimers would lead to increased agonist ILP availability, while continued secretion of heterodimers sequesters ILP antagonists. The net result would be increased IIS, and rescue of *unc-31(e928)* phenotypes. **(C)** Global deletion of *daf-2b* will remove both DAF-2 BB homodimers and AB heterodimers and therefore will have no net effect on the balance between ILP agonists and antagonists, and therefore no effect on IIS or *unc-31(e928)* phenotypes. The diagrams were created in BioRender. Gill, M. (2026) https://BioRender.com/5hynawv.

Based on our data, we conclude that reduced IIS in *unc-31* mutants is a consequence of both reduced agonist ILP secretion and a relative excess of antagonist ILPs (Fig 12A). In addition, we have determined that *unc-31* mutants not only secrete DAF-2B homodimers but they also express DAF-2A/C - DAF-2B heterodimers. Differences in the composition of the ligand binding domains of these different DAF-2 dimers could result in different affinities for agonist and antagonist ILPs. The genetic interaction between the KDEL mutant and *ins-18* deletion with respect to dauer formation suggests that DAF-2B homodimers are preferentially sequestering ILP agonists. By extension, it is tempting to speculate that DAF-2A/C - DAF-2B heterodimers may preferentially bind ILP antagonists in *unc-31* mutants (Fig 12B). In contrast, the absence of any effect of global deletion of *daf-2b* in *jluSi3; daf-2(jlu1); unc-31(e928)* could be explained by the complete absence of both DAF-2B homodimers and DAF-2B heterodimers. This would lead to no net change in the relative levels of agonist and antagonist ILP activity, and thus, no change in IIS or related phenotypes (Fig 12C).

In this study, we set out to identify regulators of DAF-2B expression and identified a mutation in the *unc-31* gene as the cause of increased DAF-2B::mScarlet in the nervous system. We have determined that this neuronal signal is derived in part from the existence of DAF-2A/C - DAF-2B hybrid receptors, which have not previously been documented *in vivo* and represent a new class of DAF-2 decoy receptors in *C. elegans*. In addition, we have determined that neuronal DAF-2B::mScarlet, either as a homodimer or a heterodimer, undergoes endocytosis in the nervous system. We speculate that an increase in DAF-2B homodimers in *unc-31* mutants, as well as in agonist ILP deficiency, functions to reinforce reduced IIS by sequestering and clearing agonist ILPs by AP2-dependent endocytosis. DAF-2B heterodimers may also undergo endocytosis and whether they function to clear ILP antagonists remains to be determined.

## Materials & methods

### Strains and maintenance

*C. elegans* strains were maintained as previously described [23]. Bristol N2 (wild-type), CB246[*unc-64(e246)*], CB928[*unc-31(e928)*], DV3805[*reSi7*]*,* MAH677[*sqIs71; sid-1(qt9)*], MQD1661[*daf-2(hq61)*]*,* MQD2428[*daf-2(hq363)*]*,* VC1218[*ins-18(ok1672)*], and ZM7963[*hpDf761*; *daf-28(tm2308)*] were obtained from the *Caenorhabditis* Genetics Center (University of Minnesota, MN). All worm strains used in the study are listed in S1 File.

### Mutagenesis of *C. elegans* and screening of mutant animal populations

Approximately 1000 MGL367[*daf-2(jlu2[daf-2b::mScarlet*])] day 1 adult animals were treated with 50 mM ethyl methanesulfonate (EMS) for 4 hours in S-basal buffer followed by washing. 100 animals were placed onto each of 10 150 mm petri dishes seeded with 5x concentrated *E.coli* OP50 bacteria to recover and lay eggs for 24 h followed by removal. These 10 populations were subsequently maintained separately throughout the remainder of the screen. 3 days later, each plate was subjected to hypochlorite treatment to extract synchronized embryo populations. Approximately 20,000 F2 embryos were extracted from each population for analysis as day 1 adults. Animals were screened for increased DAF-2B::mScarlet fluorescence in either the nervous system or coelomocytes using a fluorescence stereomicroscope. Animals of interest were isolated for further investigation. The *daf-2(jlu2); jlu22* isolate was obtained from this screen and, following whole genome sequencing, was designated MGL420[*daf-2(jlu2); unc-31(jlu22)]*.

### Plasmids

All plasmids used in the study are listed in S2 File and oligos are listed in S3 File. FRET control plasmids were constructed as follows. A *NotI-mScarlet-NheI* fragment derived from pJW2098 (oligos 1 & 2) was cloned into pMGL120 to generate pMGL279[*rab-3p::AgeI-NotI-mScarlet::unc-54 3’UTR*]. The *rab-3* 3’UTR was amplified from genomic DNA as an NheI/BsiWI fragment (oligos 3 & 4) and cloned into pMGL279 to generate pMGL280[*rab-3p::AgeI-NotI-mScarlet::rab-3 3’UTR*]. *rab-3p::AgeI-NotI-mScarlet::rab-3 3’UTR* from pMGL280 was cloned into pDD356 and pDD357 using SphI and BsiWI to replace *sun-1p::TIR1::mTagBFP2 C1::tbb-2 3’UTR*, generating pMGL281[*SEC rab-3p::AgeI-NotI-mScarlet::rab-3 3’UTR LGI*] and pMGL282[*SEC rab-3p::AgeI-NotI-mScarlet::rab-3 3’UTR LGII*] respectively.

For the monomeric mScarlet FRET control plasmid, an mScarlet fragment was amplified from pJW2098 with oligos 5 and 6 and cloned by HiFi assembly into pMGL281 cut with AgeI and NheI to generate pMGL289[*SEC rab-3p:mScarlet::rab-3 3’UTR LGI*]. For dimeric FRET control plasmids, *AgeI-mNeonGreen(no stop)-NotI* and *NotI-30aa-linker::mNeonGreen-NheI* fragments were synthesized by Genscript (pMGL278 and pMGL277 respectively) using the mNeonGreen sequence from pJW2171 and cloned into pMGL289 cut with AgeI/NheI to remove mScarlet. This resulted in the plasmid pMGL285[*rab-3p::mNeonGreen::30aa-linker::mNeonGreen::rab-3 3’UTR*]. To generate a tandem mNeonGreen mScarlet expression construct, the *NotI-30aa-linker::mNeonGreen-NheI* fragment in pMGL285 was replaced with *NotI-30aa-linker::mScarlet-NheI* derived from pJW2098 (oligos 7&2). This generated pMGL296[*SEC rab-3p::mNeonGreen::30aalinker-mScarlet::rab-3 3’UTR LGII*].For dimeric mScarlet, an *AgeI-mNeonGreen-NotI* fragment from pMGL296 was replaced with an mScarlet fragment derived from pJW2098 using oligos 5 and 8 to create pMGL308[*SEC rab-3p::mScarlet::30aalinker-mScarlet::rab-3 3’UTR LGII*].

To generate N-terminal mNeonGreen translational reporters for *rab-11.1, rab-7* and *rab-5,* each genomic coding sequence was amplified with oligos that added 5’ and 3’ overhangs for HiFi assembly (*rab-11.1* oligos 9 & 10; *rab-7* oligos 11 & 12; *rab-5* oligos 13 & 14). An *mNeonGreen::30aa-linker* fragment specific for each *rab* was amplified from pMGL285 (*mNeonGreen::linker::rab-11.1* oligos 15 & 16; *mNeonGreen::linker::rab-7* oligos 15 & 17; *mNeonGreen::linker::rab-5* oligos 15 & 18). HiFi assembly of the *rab* genomic fragment and the corresponding *mNeonGreen::linker* into pMGL282[*SEC rab-3p::mScarlet::rab-3 3’UTR LGII*] cut with AgeI-NheI resulted in the following plasmids pMGL321[*SEC rab-3p::mNeonGreen::linker::rab-11.1::rab-3 3’UTR LGII*], pMGL322[*SEC rab-3p::mNeonGreen::linker::rab-7::rab-3 3’UTR LGII*], pMGL323[*SEC rab-3p::mNeonGreen::30aa linker::rab-5::rab-3 3’UTR LGII*],

To generate a DAF-2B::mScarlet expression plasmid, mScarlet was amplified from pJW2098 as a NotI / NheI fragment with oligos 1 & 2, and a *daf-2b* cDNA without a stop codon was cloned as an AgeI / NotI fragment into a plasmid containing 3 kb *daf-2* promoter to generate pMGL258[*daf-2p::daf-2b cDNA::mScarlet*]. To localize DAF-2B::mScarlet to the ER, mScarlet was amplified from pJW2098 as a *NotI-mScarlet::KDEL-NheI* fragment with oligos 1 & 19 and cloned into pMGL258 to generate pMGL259[*daf-2p::daf-2b cDNA::mScarlet*::*KDEL*]. To generate a GFP ER marker, GFP was amplified from pJW2086 using a forward primer that added an 5’ AgeI restriction site and 57 bp corresponding to the 19 amino acid signal peptide (SP) from *hsp-3* [59] (oligo 20) and a reverse primer that added a 3’ KDEL sequence and an NheI site (oligo 21). This fragment was cloned into pMGL258 to generate pMGL271[*daf-2p::SP::GFP::KDEL::unc-54 3’UTR*].

### Transgenic strains

Strains over expressing DAF-28 and INS-18 were generated by microinjection of the plasmids pMGL219(*daf-28p::daf-28::unc-54* 3’UTR) and pMGL221(*ins-18p::ins-18::unc-54* 3’UTR) at 10 ng/μL with a *myo-3p*::GFP coinjection marker at a concentration of 50 ng/μL. 2–3 independent lines were analyzed for each construct in each background. Strains expressing DAF-2B::mScarlet with a GFP ER marker both from a 3 kb *daf-2* promoter were generated by microinjection of plasmids pMGL258[*daf-2p::daf-2b::mScarlet::unc-54* 3’UTR] and pMGL271[*daf-2p::SP::GFP::kdel::unc-54* 3’UTR] at 10 ng/µl with 50 ng/µl *rol-6* coinjection marker. DAF-2B::mScarlet::KDEL strains were generated in a similar manner using pMGL259[*daf-2p::daf-2b::mSc::kdel::unc-54* 3’UTR] and pMGL271*[daf-2p::SP::GFP::kdel::unc-54* 3’UTR]. 2–3 independent lines were analyzed for each construct in each background.

### CRISPR/Cas9 gene editing

To generate *unc-31(jlu43)* (S1 Fig)*,* we used the ribonucleoprotein method [60] but without *dpy-10* co-CRISPR. An *unc-31(jlu43)* crRNA targeting a protospacer adjacent motif (PAM) sequence close to the edit site (oligo 22) was identified using Wormbase [61]. A single stranded DNA *unc-31(jlu43)* repair template (oligo 23) containing the premature stop codon, as well as a silent mutation in the PAM site, was synthesized by Eurofins. Cas9, crRNA, and trans-activating crRNA (tracrRNA) were obtained from Integrated DNA Technologies (IDT). The injection mix was prepared according to Wang et al. [62]. Approximately 30 P0 animals were injected and singled onto individual plates. F2 *unc-31* animals were identified by their Unc phenotype. After onset of egg-laying, F2 animals were lysed, and the *unc-31* locus was amplified using oligos 24 and 25 and sequenced with oligo 26. One line with the correct edit was isolated and outcrossed twice to MGL367[*daf-2(jlu2)]* to yield MGL616[*daf-2(jlu2); unc-31(jlu43)].*

For the following CRISPR / Cas9 genome edits we used the *dpy-10* co-CRISPR approach [60]. To introduce DAF-2B::mScarlet into the *daf-2b* genomic locus of MQD1661[*daf-2(hq61)*] and MQD2428[*daf-2(hq363)*] we used CRISPR Cas9 genome editing as previously described [5]. After confirming the correct insertion and 2x outcrossing we obtained MGL660[*daf-2(jlu48[daf-2b::mScarlet daf-2a/c::mNeonGreen])* and MGL663[*daf-2(jlu49[daf-2b::mScarlet daf-2a/c::AID::mNeonGreen)*].

To convert TIR1 strains to the AID2 system [63], we used CRISPR to engineer the F79G mutation into DV3805. A crRNA close to the edit site was identified (oligo 27) and ssDNA repair template containing a silent mutation in the PAM site also introduced a BsrBI restriction site (oligo 28). PCR amplification using oligos 29 & 30 followed by BsrBI digestion was used to identify the correct mutation. After sequencing of F2 lines, one line was selected and outcrossed to N2 5 times using oligos 31–33 for genotyping, to derive MGL595[*jluSi11 [rgef-1p*::TIR1(F79G)::F2A::mTagBFP2::AID*::NLS::tbb-2 3’UTR]

The FRET control strains were generated using a modification of the protocol in Malaiwong et al. [64] to generate the CRISPR injection mix. The crRNA and repair template SEC plasmids at 10 ng/µL were injected with 2.5 ng/µL pCFJ90[*myo-2p::mcherry*], 5 ng/µL pCFJ104[*myo-3p::mcherry*] coinjection marker plasmids. pMGL289 was inserted into chromosome I with a ttTi4348 crRNA (oligo 34) and a ttTi5605 crRNA (oligo 35) was used to insert pMGL296 and pMGL308 into a chromosome II safe harbor site. 3 days post injection, 200 µL of 5 mg/ml hygromycin B (Gold Bio) was added to the injection plate (35 mm plates contained 4 mL of NGM). F2 rollers with no mCherry were identified and single picked and confirmed as homozygous as F3s. The SEC was removed by heatshock as previously described [65]. In this way we derived MGL873[*jluSi32(rab-3p;:mNeonGreen::30-aa linker::mScarlet::rab-3 3’UTR*) II], MGL874[*jluSi33(rab-3p:::mScarlet::rab-3 3’UTR)* I] and MGL875[*jluSi34(rab-3p::mScarlet::30aa-linker::mScarlet::rab-3 3’UTR*) II].

To insert mNeonGreen::AID into the genomic *apa-2* locus we generated a PCR repair template by amplifying *30aa-linker::mNeonGreen::AID* from pJW2171 using oligos 36 & 37. An *apa-2* crRNA (oligo 38) and the repair template was injected with a *rol-6* coinjection marker according to the method of [66]. The correct insertion was confirmed by PCR and sequencing using oligos 39–41, resulting in the strain MGL798[*apa-2(jlu71[apa-2::mNeonGreen::AID*])].

Strains expressing mNeonGreen::RAB-5, mNeonGreen::RAB-7 and mNeonGreen::RAB-11.1 were generated by microinjection of the plasmids pMGL323, pMGL322 and pMGL321 at a concentration of 10ng/µL with 2.5ng/µL pCFJ90[*myo-2p::mcherry*], 5ng/µL pCFJ104[*myo-3p::mcherry*] coinjection marker plasmids with ttTi5605 crRNA as described above for the FRET control plasmid. Selection was performed as per the FRET control plasmids described above and the following strains were derived - MGL726[*jluSi21(SEC rab- 3p::mNeonGreen::linker::rab-5::rab-3 3’UTR LoxN sqt-1(d) hsp::CRE HygR LoxN*]), MGL727[*jluSi22(SEC rab-3p::mNeonGreen::linker::rab-7::rab-3 3’UTR*] *LoxN sqt-1(d) hsp::CRE HygR LoxN*), MGL728[*jluSi23(SEC rab-3p::mNeonGreen::linker::rab-11.1::rab-3 3’UTR LoxN sqt-1(d) hsp::CRE HygR LoxN*]).

To generate the *daf-2b::*KDEL mutant we used a *daf-2b* crRNA as previously described [5] and a single stranded DNA repair template (oligo 42) that introduces an AluI restriction site. PCR amplification using oligos 43 & 44 followed by AluI digestion was used to identify the correct mutation. After sequencing of F2 lines, one line was selected and outcrossed to N2 2 times to derive MGL673[*daf-2(jlu44[daf-2b::kdel]*)].

To examine the effect of DAF-2B::KDEL on the subcellular localization of DAF-2A/C::mNeonGreen we used CRISPR / Cas9 to insert an *SL2::SP::mScarlet::KDEL* sequence into the 3’ end of the *daf-2a/c::AID::mNeonGreen* sequence in MQD2428[*daf-2(hq363[daf-2a/c::degron::mNeonGreen]) III].* We first sequenced the 3’ sequence of *daf-2(hq363)* (oligos 45 & 46) and identified a crRNA site in the 3’UTR of *daf-2* (oligo 47). A repair template consisting of the SL2 leader sequence, the signal peptide (SP) sequence from *hsp-3* [59], and mScarlet KDEL was synthesized by Genscript and amplified from pMGL297 with oligos 48 & 49. The crRNA and repair template were injected into *daf-2(hq363)* using the *dpy-10* co-CRISPR approach [60]. The correct insertion was confirmed using oligos 50–52, generating the strain MGL762[*daf-2(jlu56[daf-2a/c::AID::mNeonGreen::SL2::SP::mScarlet::KDEL*]). We then introduced the KDEL epitope into the *daf-2b* locus of MGL762 as described above to generate MGL781[*daf-2(jlu64[daf-2b::KDEL daf-2a/c::AID::mNeonGreen::SL2::SP::mScarlet::KDEL*]).

### Genetic crosses

Genetic crosses were performed using standard methods and strains made in this study are listed in S1 File. In all cases, strains crossed into MGL367[*daf-2(jlu2[daf-2b::*mScarlet])] were screened for fluorescence, and strains crossed into *unc-31* and *unc-64* mutants were screened for the Unc phenotype. Strains crossed into *hpDf761*; *daf-28(tm2308)* were screened for Daf-c at 20 degrees followed by genotyping.

Complementation was performed by first crossing MGL420[*daf-2(jlu2); unc-31(jlu22)]* to N2 and isolating Unc animals without DAF-2B::mScarlet to generate MGL422[*unc-31(jlu22)*]. CB246[*unc-64(e246)*] and CB928[*unc-31(e928)*] hermaphrodites were mated with N2 males to produce heterozygous *unc-64* and *unc-31* males. These F1 males were then crossed into MGL422[*unc-31(jlu22)*] hermaphrodites and dauer formation was scored in the F2 progeny.

CB928[*unc-31(e928)*] was crossed into MGL367[*daf-2(jlu2)*] to produce MGL434[*daf-2(jlu2); unc-31(e928)*]. VC1218[*ins-18(ok1672)*] was crossed into MGL434[*daf-2(jlu2); unc-31(e928)*] to derive MGL626[*daf-2(jlu2)*], MGL627[*ins-18(ok1672); daf-2(jlu2)*], MGL628[*daf-2(jlu2); unc-31(e928)*], MGL629[*ins-18(ok1672); daf-2(jlu2); unc-31(e928)*], MGL634[*unc-31(e928)*] and MGL635[*ins-18(ok1672); unc-31(e928)*] from a single cross. The *ins-18(ok1672)* allele was genotyped with oligos 53–54. MGL367[*daf-2(jlu2)*] was crossed into ZM7963[*hpDf761; daf-28(tm2308)*] to derive MGL630[*daf-2(jlu2)*], MGL631[*daf-2(jlu2); daf-28(tm2308)*], MGL632[*hpDf761; daf-2(jlu2)*], and MGL633[*hpDf761; daf-2(jlu2); daf-28(tm2308)*] from a single cross. The *daf-28(tm2308)* allele was genotyped with oligos 55 & 56 and the *hpDf761* deletion was assessed with oligos 57–59.

*unc-31(e928)* was crossed into MGL660[*daf-2(jlu48[daf-2b::mScarlet daf-2a/c::mNeonGreen])]* to generate MGL661[*daf-2(jlu48); unc-31(e928)].* MGL595[*jluSi11 [rgef-1p*::TIR1(F79G)::F2A::mTagBFP2::AID*::NLS::*tbb-2* 3’UTR] was crossed into MGL663[*daf-2(jlu49[daf-2b::mScarlet daf-2a/c::AID::mNeonGreen)]* to generate MGL666*[jluSi11; daf-2(jlu49)]* which was subsequently crossed into *unc-31(e928)* to generate MGL669*[jluSi11; daf-2(jlu49); unc-31(e928)]* and *hpDf761*; *daf-28(tm2308)* to generate MGL714[*jluSi11; hpDf761; daf-2(jlu49); daf-28(tm2308)].* The TIR1 insertion was genotyped using oligos 31–33 and *daf-2(jlu49)* was genotyped using oligos 60–62.

MAH677[*sqIs71; sid-1(qt9)*] was crossed with MGL367[*daf-2(jlu2)*] to generate MGL751[*daf-2(jlu2) sqIs71; sid-1(qt9*)]. During this cross it was determined that *sqIs71* is integrated on chromosome III, approximately 30 map units from *daf-2.* MAH677[*sqIs71; sid-1(qt9)*] was crossed with CB928[*unc-31(e928)*] to generate MGL764[*sqIs71; unc-31(e928*); *sid-1(qt9)*]. MGL751[*daf-2(jlu2) sqIs71; sid-1(qt9)*] was crossed with MGL764[*sqIs71; unc-31(e928*); *sid-1(qt9)*] to generate MGL752[*daf-2(jlu2) sqIs71; unc-31(e928); sid-1(qt9*)]. The *sid-1(qt9)* allele was genotyped with oligos 63 & 64 and *sqIs71* was monitored by observing GFP. *daf-2(jlu2)* was genotyped using oligos 51, 65, 66.

MGL798[*apa-2(jlu71[apa-2::mNeonGreen::AID*]) was crossed with MGL595[jluSi11[rgef-1p::TIR1(F79G)::F2A::mTagBFP2::AID*::NLS::tbb-2 3’UTR] to generate MGL831[*jluSi11; apa-2(jlu71)*]. MGL831 was crossed with MGL434[*daf-2(jlu2); unc-31(e928)*] to generate MGL834[*jluSi11; daf-2(jlu2); apa-2(jlu71)*] and MGL835[*jluSi11; daf-2(jlu2); unc-31(e928); apa-2(jlu71)*]. Genotyping was performed using oligos 39–41.

MGL726[*jluSi21(SEC rab-3p::mNeonGreen::linker::rab-5::rab-3 3’UTR LoxN sqt-1(d) hsp::CRE HygR LoxN*)], MGL727[*jluSi22(SEC rab-3p::mNeonGreen::linker::rab-7::rab-3 3’UTR LoxN sqt-1(d) hsp::CRE HygR LoxN*)] and MGL728[*jluSi23(SEC rab-3p::mNeonGreen::linker::rab-11.1::rab-3 3’UTR LoxN sqt-1(d) hsp::CRE HygR LoxN)*] were crossed with MGL434[*daf-2(jlu2); unc-31(e928)*] to generate MGL774[*jluSi21; daf-2(jlu2); unc-31(e928)*], MGL775[*jluSi22; daf-2(jlu2); unc-31(e928)*] and MGL776[*jluSi23; daf-2(jlu2); unc-31(e928)]* respectively*.* Homozygosity of the *mNeonGreen::rab* transgenes was achieved by monitoring mNeonGreen fluorescence and the Rol phenotype.

We previously generated a *daf-2b* deletion strain, MGL302[*jluSi3; daf-2(jlu1)*]*,* in which *daf-2(jlu1)* is a *daf-2bc* deletion and *jluSi3* is a single copy insertion of *daf-2c* cDNA at the ttTi5605 Mos insertion locus that rescues the *daf-2c* deletion [5]. We crossed MGL302[*jluSi3; daf-2(jlu1)*] into CB928[*unc-31(e928)*] to derive MGL568[*jluSi3*], MGL567[*jluSi3*; *daf-2(jlu1)*], MGL566[*jluSi3*; *unc-31(e928)*], and MGL565[*jluSi3*; *daf-2(jlu1); unc-31(e928)]*. These *daf-2b* deletion strains were genotyped using oligos 67–71.

MGL673*[daf-2(jlu44[daf-2b::kdel])* was crossed into *unc-31(e928)* to generate MGL675[*daf-2(jlu44); unc-31(e928)*]. Genotyping was performed using oligos 43 and 44, followed by restriction digest. MGL675 was subsequently crossed to VC1218 to produce MGL715[*unc-31(e928)*], MGL716[*ins-18(ok1672); unc-31(e928)*], MGL717[*daf-2(jlu44); unc-31(e928)*], and MGL718[*ins-18(ok1672); daf-2(jlu44); unc-31(e928)*].

### RNA interference

RNAi clones were obtained from the Open Biosystems RNAi library (*rab-5, rab-11.1, aps-2, dpy-23)* or the Ahringer library (*rab-7, apa-2, daf-16*) and sequenced to confirm their identity (oligos 72 & 73). RNAi bacteria were grown in the presence of tetracycline (5 µg/mL) and carbenicillin (50 µg/mL) antibiotics, and single colonies were inoculated in LB with carbenicillin (50 µg/mL) for 16–20 h at 37°C. Nematode growth medium (NGM) plates containing 1 mM IPTG and 50 µg/ mL carbenicillin were seeded with an overnight culture of bacteria, dried in a sterile hood, and maintained at room temperature overnight. Ten to fifteen gravid adults were transferred to fresh RNAi plates, allowed to lay eggs for 2 h, and removed. The L4440 empty vector was used as our negative control. Animals were assayed three days later by fluorescence microscopy. For neuron-specific RNAi experiments animals were examined in the second generation of exposure to RNAi and for systemic RNAi animals were placed on RNAi as eggs and analyzed as D1 adults.

### Fluorescence imaging and quantitation

Images were obtained using a Hamamatsu ORCA-Fusion BT camera on a Nikon Eclipse Ti2-E inverted microscope with Nikon CFI Plan APO 𝛌D 40X or 60x(oil) objectives, illuminated with a multichannel Nikon D-LEDI fluorescence LED illumination system which maintains constant intensity and light source alignment. Images were compiled and analyzed with Nikon NIS Elements software. Within each experiment, images for different genetic conditions were taken with identical LED intensity, exposure times and camera settings. For nervous system expression of DAF-2B::mScarlet in day 1 adults, Nikon nd.2 images were analyzed by selecting the region between the back of the metacorpus of the pharynx to the mid-section of the terminal bulb (to avoid interference from intestinal autofluorescence). The average pixel intensity of the area was compared between groups with background correction (expressed as Arbitrary Units, A.U.). Analysis of *C. elegans* coelomocytes was performed by imaging anterior coelomocytes not occluded by the germline or intestine. Z-stack image series were taken at 1 µm intervals and maximum intensity projections were derived. Regions of interest were determined by thresholding using Otsu’s method and measured using integrated pixel density in FIJI. For colocalization experiments, head regions were imaged using different exposures for fluorescence balance between channels. Z-planes of 1.5-2 µm intervals were taken, followed by deconvolution using NIS Elements and thresholding based on the background. All representative images were generated using a pseudocolor merge function, consistent thresholding based upon the background, and deconvolution projection through the NIS Elements.

### Live *in vivo* Fluorescence Lifetime Imaging (FLIM) FRET

*Microscope and optics:* Fluorescence lifetime imaging (FLIM) was performed on a Leica STELLARIS 8 FALCON confocal microscope (Leica Microsystems) equipped with a tunable White Light Laser (WLL, 440–790 nm, 80 MHz) and HyD S photon-counting detectors controlled by LAS X software. A HC FLUOTAR L VISIR 25x/0.95 NA water immersion/1.40 NA objective was used unless noted.

*Excitation, detection and acquisition:* mNeonGreen and mScarlet were excited at λ_ex,mNg_ = 490 nm and λ_ex,mSc_ = 570 nm, respectively, from the WLL. Emission for both channels were collected on separate HyD S detectors with a λ_em,mNg_ = 510–550 nm and λ_em,mSc_ = 590–650 nm bandpass filters. Laser power was adjusted to maintain per-pixel count rates below 1-photon per pulse rate to avoid pulse-pileup error. Image size was 512 × 512 pixels with pinhole set to 1.0 AU with scan speed of 400 for a resulting pixel dwell of 2–3 µs. A 3.0 zoom-factor was applied to each image resulting in a voxel size of 289 nm. In total, 6-line scans for 5-frames per animal were used to limit motion and photobleaching.

*Calibration and IRF:* The instrument response function (IRF) was estimated using a global IRF fit across all data sets within the Leica LAS X FLIM analysis software (with FALCON module); lifetime calibration was done with AlexaFluor568 (𝜏 = 3.5 ns) to confirm absolute scale before sample runs.

*Analysis:* Lifetime maps were generated in LAS X using 2-component exponential fitting to account for both mScarlet and a fixed autofluorescence signal. Autofluorescence was determined by imaging of N2 animals and iterative 2-component fits to determine a converged amplitude weighted FLT (𝜏_auto_ = 0.152 ns). For homoFRET, donor-only mScarlet lifetime was determined using both mScarlet monomer and a tandem mNeonGreen-linker-mScarlet control strain imaged under identical conditions. Regions of interest (ROIs) were defined on the mScarlet intensity image; pixel-wise fits were pooled per ROI.

*Quality control:* Count-rate-dependent artifacts (“pile-up”) were minimized by adjusting laser power and dwell time to maintain 1-photon per pulse; detectors were monitored to avoid overload.

### Conditional degradation using the auxin-inducible degradation system

DAF-2 protein was conditionally depleted using the auxin-inducible degradation system [67] using modified TIR1 constructs bearing an F79G base-pair substitution [63]. 5-Ph-IAA (Cayman Chemical, Cat. 38161) was dissolved in ethanol and added to NGM plates at a concentration of 10 µM. NGM with 0.1% ethanol was used as a solvent control. An overnight culture of *E. coli* OP50 was seeded on plates and allowed to dry. Subsequently, animals were placed on these plates for 5 h followed by imaging.

### Phenotypic assays

For dauer entry assays in the *unc-31* mutant background, 3.5 cm NGM plates were seeded with 100 µL of an overnight culture of *E. coli* OP50. Eggs from a 2 h lay were maintained at 26.1°C for 48–50 h. Dauers were scored on the basis of morphology and expressed as a percentage of the total population. For dauer formation using DAF-28 overexpression, 2 independent isolates were examined and data pooled. Lifespan assays were performed at 20°C on NGM plates with a fresh lawn of OP50 *E. coli* bacteria and 12.5 μg/mL 5-fluoro-2′-deoxyuridine (FUDR) starting at the first day of adulthood. Animals were transferred again on the second day of adulthood, then every 3–5 days until completion. Death was scored by loss of touch-provoked movement, and animals lost due to uterine prolapse or crawling up the side of the petri dish were censored. Entire experiments were replicated three times. Lifespan data was graphed using a Kaplan–Meier format. Egg retention phenotyping was performed as previously published [68] with modification. Briefly, late day 1 adult animals were mounted on 2% agarose pads and eggs within each uterus were counted at high magnification.

### Whole genome sequencing

Mass cultures of MGL420[*daf-2(jlu2); unc-31(jlu22)*], our original screen isolate, were grown on 100 mm NGM plates. Worms were harvested, washed with S-basal, followed by purging for 2–3 h on a rocking shaker at room temperature and subsequently washed a further 3 times. Worms were pelleted by centrifugation and snap frozen on dry ice. Frozen worm pellets (~100 µl) were sent to Azenta/GeneWiz for genomic extraction, quantitation, library preparation and sequencing.

Illumina libraries of genomic DNA were prepared and sequenced by GeneWiz (South Plainfield, NJ) to 200x coverage. Reads were trimmed using TrimGalore (0.6.0) and mapped to the BSgenome.Celegans.UCSC.ce11 (1.4.2) genome using BWA mem (0.7.17-r1188). Aligned reads were sorted with Samtools (1.16.1) and duplicates were identified using MarkDuplicates (Picard, 2.18.16). The GATK Haplotype caller (4.1.2.0) was used to identify sequence variations with respect to the reference ce11 genome. The VariantAnnotation package (1.50.0) within R version (4.4.0) was used to filter variants for homozygous, single nucleotide polymorphisms with read depth > 15 that were not present in all samples. These variants were annotated using the TxDb.Celegans.UCSC.ce11.ensGene (3.15.0) annotation package to consider all splice donor/acceptor mutations and non-synonymous protein coding mutations.

### Quantitative-RT-PCR

Quantitative RT-PCR was performed as previously described [33] with minor modifications. Populations of day 1 adult animals, synchronized by egg lay, were generated for RNA extraction. Approximately ~500 animals were harvested from 6 independent populations per strain and RNA was extracted as previously reported [69], with minor modifications. Populations were subjected to proteinase K treatment and lysis in a heating block, followed by pulse spin and a brief centrifugation (this removes eggs and debris). Lysates were immediately placed into the RLT buffer with β-mercaptoethanol from the QIAGEN RNeasy Kit. We followed manufacturers protocol to extract total RNA, which was measured with a Nanodrop spectrophotometer. 1 µg of total RNA was converted to cDNA using the Maxima H-minus cDNA Synthesis kit (ThermoFisher Scientific) which includes a dsDNAase degradation step. Samples were diluted in 10 mM Tris to 10 ng/µL. cDNA samples were analyzed by quantitative PCR using an Applied Biosystems QuantStudio 3 instrument. PCR on cDNA was performed using a PowerUp SYBR Green Master Mix (Applied Biosystems) and all annealing was performed at 60°C. All conditions were performed with 3 technical replicates. Cycle quantification (Cq) values were measured with the QuantStudio Design and Analysis software. Eight reference genes (*act-1, ama-1, cdc-42, pmp-3, iscu-1, snb-1, rps-2, rps-23*; oligos 74–89) were measured in each of our 12 samples (6 samples from each genotype) to determine which were most stable. M-analysis indicated that the most stable reference genes were *cdc-42* and *ama-1*, and 2 reference genes were sufficient. Oligos for *daf-2a, daf-2b* and *daf-2c* (oligos 90–95) were designed to span exon-exon boundaries and were evaluated for efficiency using standard curves across a large concentration range. Oligos (96 & 97) for *hsp-4* were previously reported [70]. Relative quantification (RQ) values were derived using the average cycle threshold (Ct) value of the control strain (N2) replicates. Relative gene expression was calculated using Pffafl’s formula [71] and included the geometric mean of the RQ values derived from the reference genes.

### Statistical analysis

Statistical analyses were performed on pooled replicate data using GraphPad Prism v8.0 with p < 0.05 indicating significance. All raw data and statistical analyses for individual replicates are presented in S1 File. For pairwise comparisons the two-tailed Student’s *t*-*t*est without correction was used. For comparisons k > 2, one-way ANOVA followed by a Dunnett’s post hoc test was used when comparing multiple conditions to a control only, and Sidak’s test was used for selected pair-wise comparisons. The Log-rank test was used to analyze lifespan data.

## Supporting information

S1 Fig *unc-31* mutations influence DAF-2B::mScarlet expression. **(A**) Organization of the *unc-31a* genomic locus with the locations of *unc-31* alleles identified in this study indicated, as well as the *e928* deletion. **(B)** Protein organization of UNC-31A and location of premature stop mutation. **(C)** CRISPR / Cas9 genome editing strategy to generate *unc-31(jlu43)* allele. **(D)** Representative images illustrating increased DAF-2B::mScarlet in neurons of the CRISPR allele *unc-31(jlu43)* and the null allele *unc-31(e928)*. The *daf-2(jlu2)* images are the same as in Figure 2B and quantitation is shown in Fig 2G. Panels A-C were created in BioRender. Gill, M. (2026) https://BioRender.com/5hynawv.
(TIFF)

S2 Fig *daf-2* transcripts are elevated in *unc-31* mutants. Endogenous *daf-2a, daf-2b* and *daf-2c* transcripts are elevated in *unc-31(e928)* mutants. qPCR was used to examine relative gene expression of *daf-2a, daf-2b* and *daf-2c* transcripts in wild type and *unc-31(e928)* animals. Data are derived from 6 independent populations and normalized to 2 reference genes. Student’s *t-*test * p < 0.05, *** p < 0.001, **** p < 0.0001.
(TIFF)

S3 Fig Loss of agonist ILPs increases DAF-2B homodimers. **(A)** DAF-2A/C::mNeonGreen is reduced in *jluSi11; daf-2(jlu49)* and *jluSi11; hpDf761; daf-2(jlu49); daf-2(tm2308)* animals treated with 10 µM 5-Ph-IAA. **(B)** DAF-2B::mScarlet is reduced in *jluSi11; daf-2(jlu49)* and *jluSi11; hpDf761; daf-2(jlu49); daf-2(tm2308)* animals treated with 10 µM 5-Ph-IAA. DAF-2B mScarlet is higher in *jluSi11; daf-2(jlu49)* and *jluSi11; hpDf761; daf-2(jlu49); daf-2(tm2308)* animals compared *with jluSi11; daf-2(jlu49)* both before and after auxin. *jluSi11; hpDf761; daf-2(jlu49); daf-28(tm2308)* exhibited 100% dauer arrest, but spontaneously recovered within 24 h. We therefore imaged these recovered animals as D1 adults. *jluSi11; daf-2(jlu49)* animals did not undergo dauer arrest and were imaged as D1 adults. Data are pooled from 3 biological replicates. One-way ANOVA with Sidak’s post-hoc test for indicated pair-wise comparisons: ns = not significant, * p < 0.05, **** p < 0.0001.
(TIFF)

S4 Fig FLIM-FRET confirms the existence of DAF-2B homodimers and heterodimers. **(A)** Ethanol control worms have DAF-2B::mScarlet FLT that is not different from mScarlet monomer controls, while 5-Ph-IAA treated worms have DAF-2B::mScarlet FLT that is not different from the mScarlet dimer control. **(B)** DAF-2A/C::mNeonGreen levels corresponding to the FLIM results shown in Fig 6C & 6D, demonstrating the extent of auxin-induced degradation of DAF-2. One-way ANOVA with Sidak’s post-hoc test for indicated pair-wise comparisons, ns = not significant, **** p < 0.0001.
(TIFF)

S5 Fig APA-2::mNG::AID knockdown with 5-Ph-IAA and systemic RNAi. **(A)** Representative images of neuronal knockdown of APA-2::mNeonGreen::AID after treatment with 10 µM 5-Ph-IAA for 5 h in *jluSi11; daf-2(jlu2); unc-31(e928); apa-2(jlu71)* D1 adults. **(B)** Representative images of neuronal knockdown of APA-2::mNeonGreen::AID after treatment with 10 µM 5-Ph-IAA for 24 h in *jluSi11; daf-2(jlu2); unc-31(e928); apa-2(jlu71)* D1 adults. **(C)** Neuronal APA-2::mNeonGreen::AID levels are reduced after treatment with 5-Ph-IAA for 24 h from L4 in both *jluSi11; daf-2(jlu2); apa-2(jlu71)* and *jluSi11; daf-2(jlu2); unc-31(e928); apa-2(jlu71)* backgrounds. **(D)** DAF-2B::mScarlet levels are reduced in *jluSi11; daf-2(jlu2); unc-31(e928); apa-2(jlu71)* mutants, but not in *jluSi11; daf-2(jlu2); apa-2(jlu71)* worms, after treatment with 5-PhIAA for 24 h from L4. **(E)** Systemic *apa-2* RNAi reduces expression of neuronal APA-2::mNeonGreen::AID in jluSi11; daf-2(jlu2); apa-2(jlu71). Data are pooled from 4 (C, D) or 3 (E) biological replicates. One-way ANOVA with Sidak’s post-hoc test for indicated pair-wise comparisons (C and D) and Student’s t-test (E), ns = not significant. **** p < 0.0001.
(TIFF)

S6 Fig Systemic *apa-2* RNAi affects endocytosis in agonist ILP deletion mutants. **(A)** Systemic *apa-2* RNAi reduces expression of neuronal DAF-2B::mScarlet in *jluSi11; daf-2(jlu49)* and *jluSi11; hpDf761; daf-2(jlu49); daf-2(tm2308)* day 1 adults treated with ethanol. **(B)** *apa-2* RNAi reduces expression of neuronal DAF-2B::mScarlet in *jluSi11; daf-2(jlu49)* and *jluSi11; hpDf761; daf-2(jlu49); daf-2(tm2308)* day 1 adults treated with 10 µM 5-Ph-IAA. *hpDf761; daf-2(jlu2); daf-2(tm2308)* and *jluSi11; hpDf761; daf-2(jlu49); daf-2(tm2308)* animals exhibited 100% dauer arrest, but spontaneously recovered within 24 h. We therefore imaged these recovered animals as D1 adults. *jluSi11; daf-2(jlu49)* animals did not undergo dauer arrest and were imaged as D1 adults. Data are pooled from 3 biological replicates. One-way ANOVA with Sidak’s post-hoc test for indicated pair-wise comparisons, ** p < 0.01, *** p < 0.001, **** p < 0.0001.
(TIFF)

S7 Fig Genomic organization of *daf-2b* mutants. **(A)** Organization of the wild type *daf-2* locus illustrating the splicing events that differentiate *daf-2a, daf-2b* and *daf-2c* transcripts. **(B)** In the *daf-2b* deletion mutant, *jluSi3; daf-2(jlu1),* the genomic sequence between exons 11 and 12 is removed, generating a *daf-2b / daf-2c* deletion (*jlu1)*, with *daf-2c* expression restored by a single copy *daf-2c* cDNA insertion at the ttTi5605 safe harbor site on chromosome II (*jluSi3)*. **(C)** In *daf-2(jlu44),* a short sequence encoding a KDEL ER retention motif has been added to the 3’ end of the *daf-2b* genomic locus. Created in BioRender. Gill, M. (2026) https://BioRender.com/5hynawv.
(TIFF)

S8 Fig Replicate lifespan experiments for the effect of *daf-2b* on lifespan in *unc-31(e928).* **(A)** Effect of *jluSi3; daf-2(jlu1)* on lifespan in *unc-31(e928)* mutants. **(B)** Effect of *daf-2(jlu44)* on lifespan in *unc-31(e928)* mutants. Log rank test, ns = not significant, ** p < 0.01, **** p < 0.0001
(TIFF)

S9 Fig DAF-2B::KDEL does not increase ER stress. The *daf-2b::KDEL* mutation does not change expression of the ER stress response marker, *hsp-4.* qPCR was used to examine relative gene expression of *hsp-*4 in N2 and *unc-31(e928)* backgrounds with or without *daf-2(jlu44).* Data are derived from 6 independent populations and normalized to 2 reference genes. One-way ANOVA with Sidak’s post-hoc test for indicated pair-wise comparisons: ns = not significant.
(TIFF)

S10 Fig Egg retention in *unc-31(e928)* mutants is suppressed by *daf-16* RNAi. Egg retention is reduced in *unc-31(e928)* mutants following *daf-16* RNAi. Data are pooled from 3 biological replicates. Student’s *t*-test: **** p < 0.0001.
(TIFF)

S1 File List of strains used in this study. (XLSX)

S2 File List of plasmids used in this study. (XLSX)

S3 File List of oligos used in this study. (XLSX)

S4 File All data used in main and supplemental figures. (XLSX)
