# Supplementary figures and images for: Partitioning of the truncated insulin receptor DAF-2B between homodimers and heterodimers influences insulin signaling in C. elegans
Source: PLoS Genet. 2026 Jul 23;22(7):e1012240. doi: 10.1371/journal.pgen.1012240 (PMC13421766; doi:10.1371/journal.pgen.1012240)

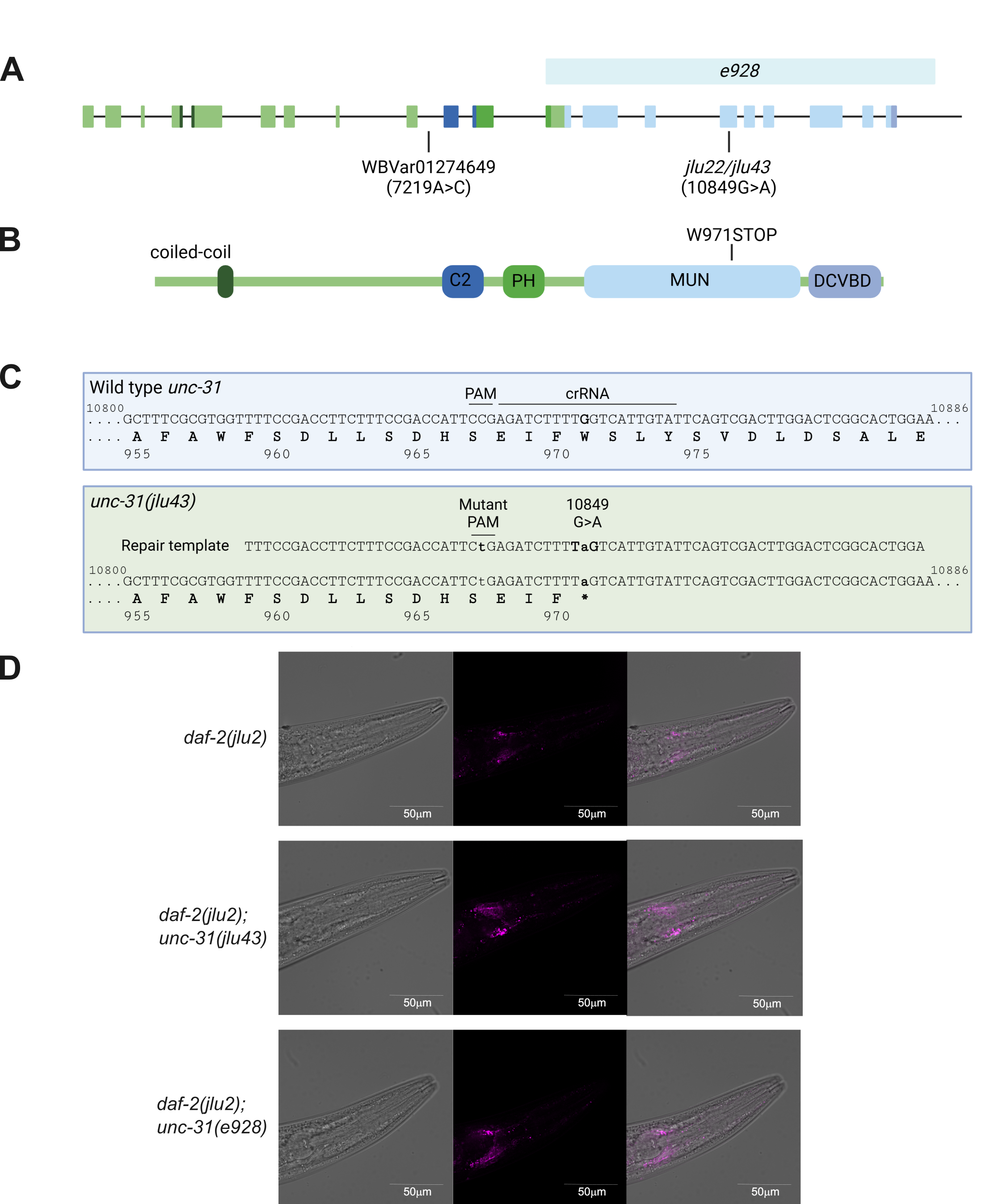

Supplement: S1 Fig — (A) Organization of the unc-31a genomic locus with the locations of unc-31 alleles identified in this study indicated, as well as the e928 deletion. (B) Protein organization of UNC-31A and location of premature stop mutation. (C) CRISPR / Cas9 genome editing strategy to generate unc-31(jlu43) allele. (D) Representative images illustrating increased DAF-2B::mScarlet in neurons of the CRISPR allele unc-31(jlu43) and the null allele unc-31(e928). The daf-2(jlu2) images are the same as in Figure 2B and quantitation is shown in Fig 2G. Panels A-C were created in BioRender. Gill, M. (2026) https://BioRender.com/5hynawv. (TIFF) [file pgen.1012240.s001.tiff]

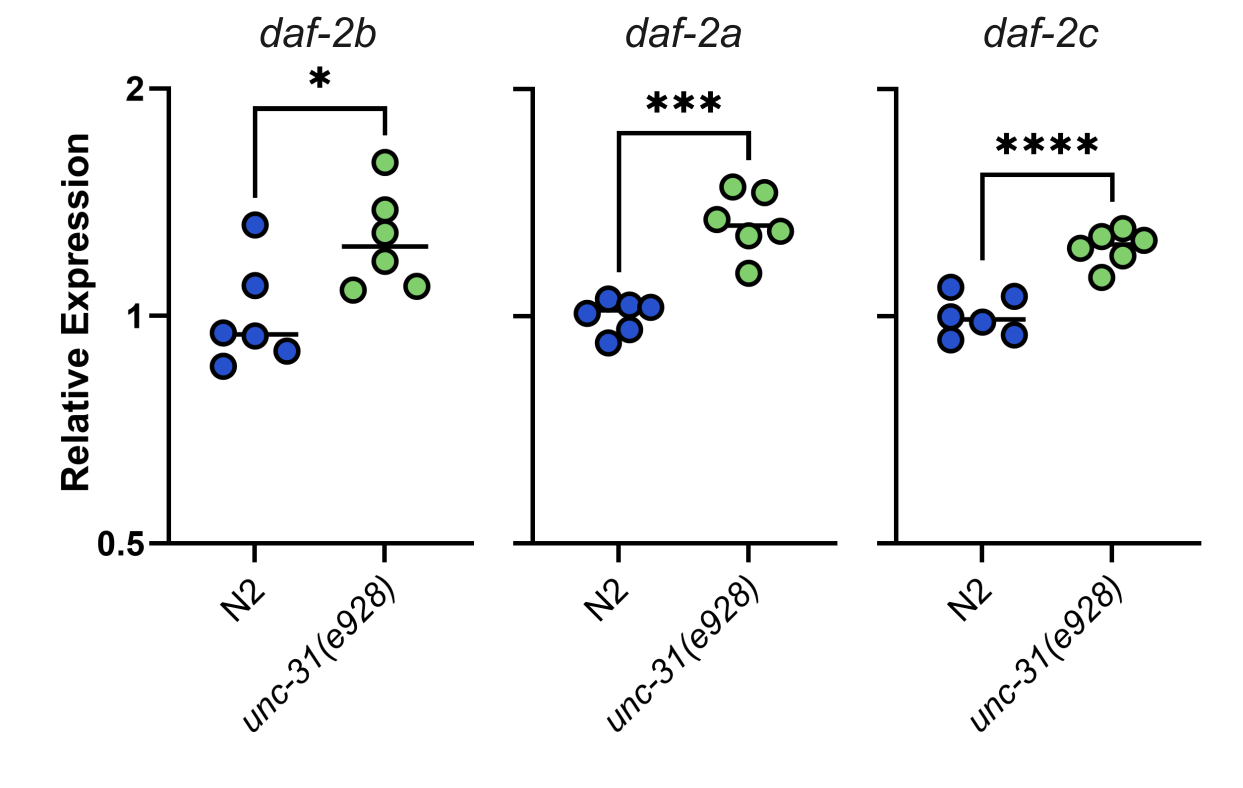

Supplement: S2 Fig — Endogenous daf-2a, daf-2b and daf-2c transcripts are elevated in unc-31(e928) mutants. qPCR was used to examine relative gene expression of daf-2a, daf-2b and daf-2c transcripts in wild type and unc-31(e928) animals. Data are derived from 6 independent populations and normalized to 2 reference genes. Student’s t-test * p < 0.05, *** p < 0.001, **** p < 0.0001. (TIFF) [file pgen.1012240.s002.tiff]

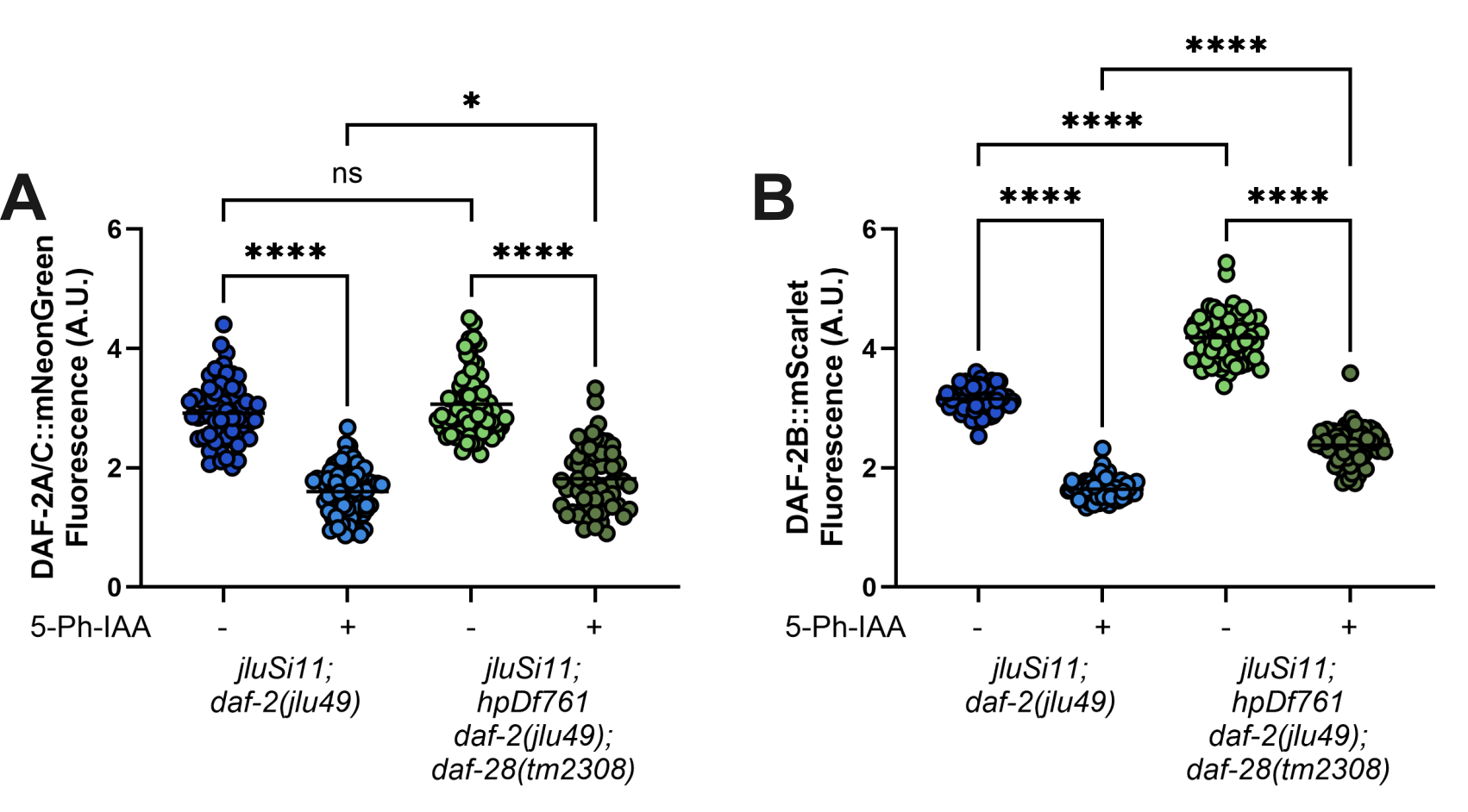

Supplement: S3 Fig — (A) DAF-2A/C::mNeonGreen is reduced in jluSi11; daf-2(jlu49) and jluSi11; hpDf761; daf-2(jlu49); daf-2(tm2308) animals treated with 10 µM 5-Ph-IAA. (B) DAF-2B::mScarlet is reduced in jluSi11; daf-2(jlu49) and jluSi11; hpDf761; daf-2(jlu49); daf-2(tm2308) animals treated with 10 µM 5-Ph-IAA. DAF-2B mScarlet is higher in jluSi11; daf-2(jlu49) and jluSi11; hpDf761; daf-2(jlu49); daf-2(tm2308) animals compared with jluSi11; daf-2(jlu49) both before and after auxin. jluSi11; hpDf761; daf-2(jlu49); daf-28(tm2308) exhibited 100% dauer arrest, but spontaneously recovered within 24 h. We therefore imaged these recovered animals as D1 adults. jluSi11; daf-2(jlu49) animals did not undergo dauer arrest and were imaged as D1 adults. Data are pooled from 3 biological replicates. One-way ANOVA with Sidak’s post-hoc test for indicated pair-wise comparisons: ns = not significant, * p < 0.05, **** p < 0.0001. (TIFF) [file pgen.1012240.s003.tiff]

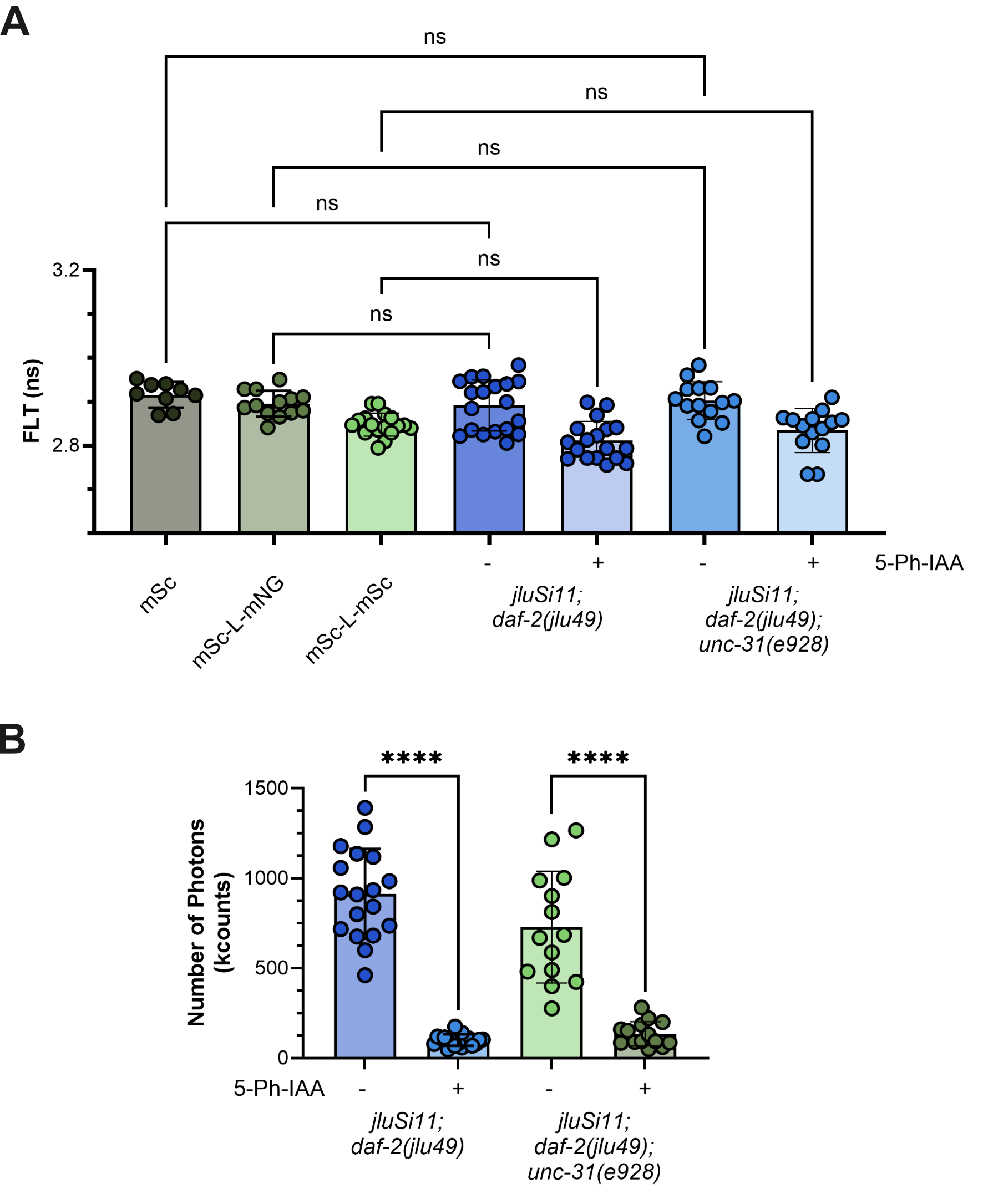

Supplement: S4 Fig — (A) Ethanol control worms have DAF-2B::mScarlet FLT that is not different from mScarlet monomer controls, while 5-Ph-IAA treated worms have DAF-2B::mScarlet FLT that is not different from the mScarlet dimer control. (B) DAF-2A/C::mNeonGreen levels corresponding to the FLIM results shown in Fig 6C & 6D, demonstrating the extent of auxin-induced degradation of DAF-2. One-way ANOVA with Sidak’s post-hoc test for indicated pair-wise comparisons, ns = not significant, **** p < 0.0001. (TIFF) [file pgen.1012240.s004.tiff]

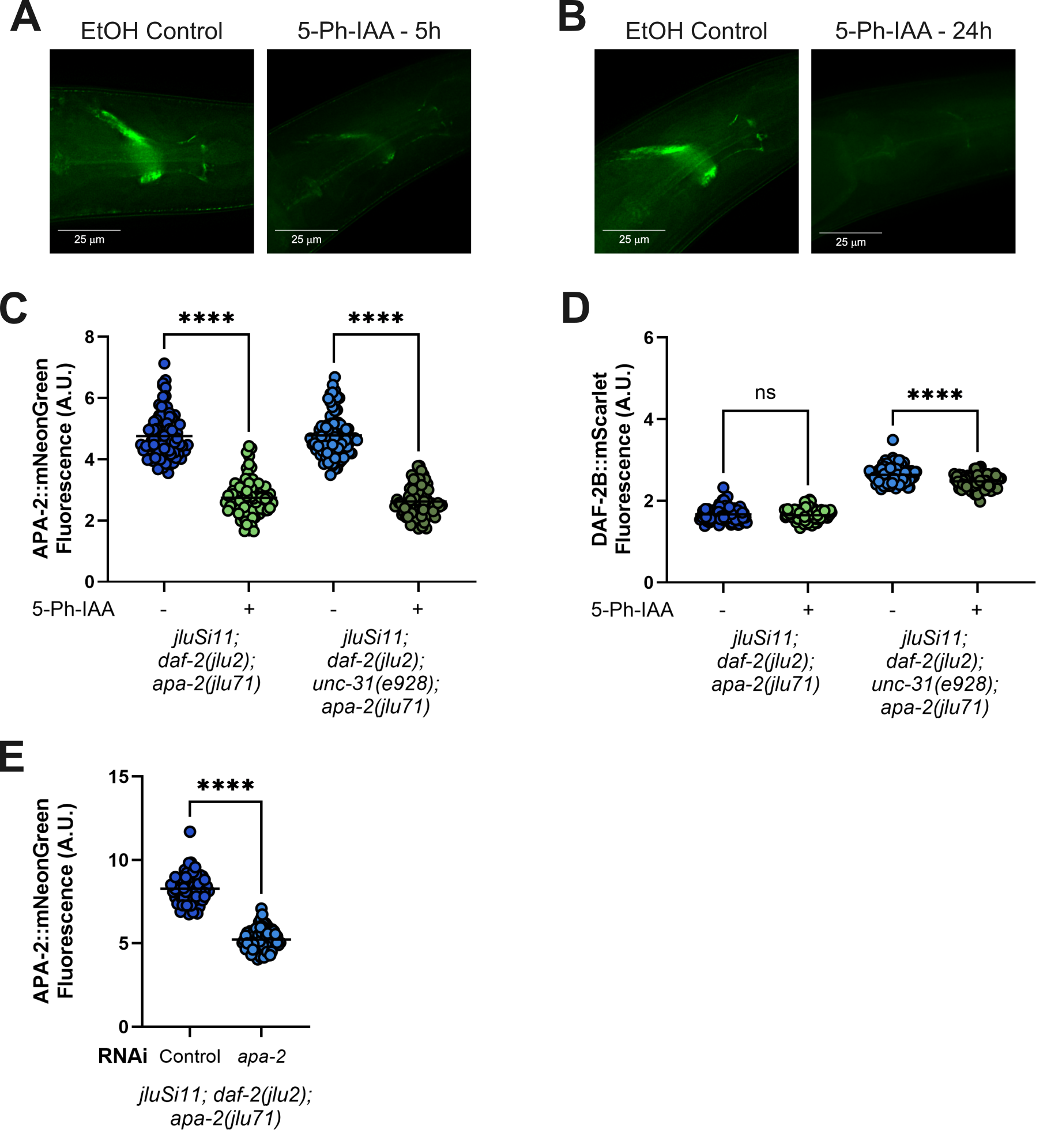

Supplement: S5 Fig — (A) Representative images of neuronal knockdown of APA-2::mNeonGreen::AID after treatment with 10 µM 5-Ph-IAA for 5 h in jluSi11; daf-2(jlu2); unc-31(e928); apa-2(jlu71) D1 adults. (B) Representative images of neuronal knockdown of APA-2::mNeonGreen::AID after treatment with 10 µM 5-Ph-IAA for 24 h in jluSi11; daf-2(jlu2); unc-31(e928); apa-2(jlu71) D1 adults. (C) Neuronal APA-2::mNeonGreen::AID levels are reduced after treatment with 5-Ph-IAA for 24 h from L4 in both jluSi11; daf-2(jlu2); apa-2(jlu71) and jluSi11; daf-2(jlu2); unc-31(e928); apa-2(jlu71) backgrounds. (D) DAF-2B::mScarlet levels are reduced in jluSi11; daf-2(jlu2); unc-31(e928); apa-2(jlu71) mutants, but not in jluSi11; daf-2(jlu2); apa-2(jlu71) worms, after treatment with 5-PhIAA for 24 h from L4. (E) Systemic apa-2 RNAi reduces expression of neuronal APA-2::mNeonGreen::AID in jluSi11; daf-2(jlu2); apa-2(jlu71). Data are pooled from 4 (C, D) or 3 (E) biological replicates. One-way ANOVA with Sidak’s post-hoc test for indicated pair-wise comparisons (C and D) and Student’s t-test (E), ns = not significant. **** p < 0.0001. (TIFF) [file pgen.1012240.s005.tiff]

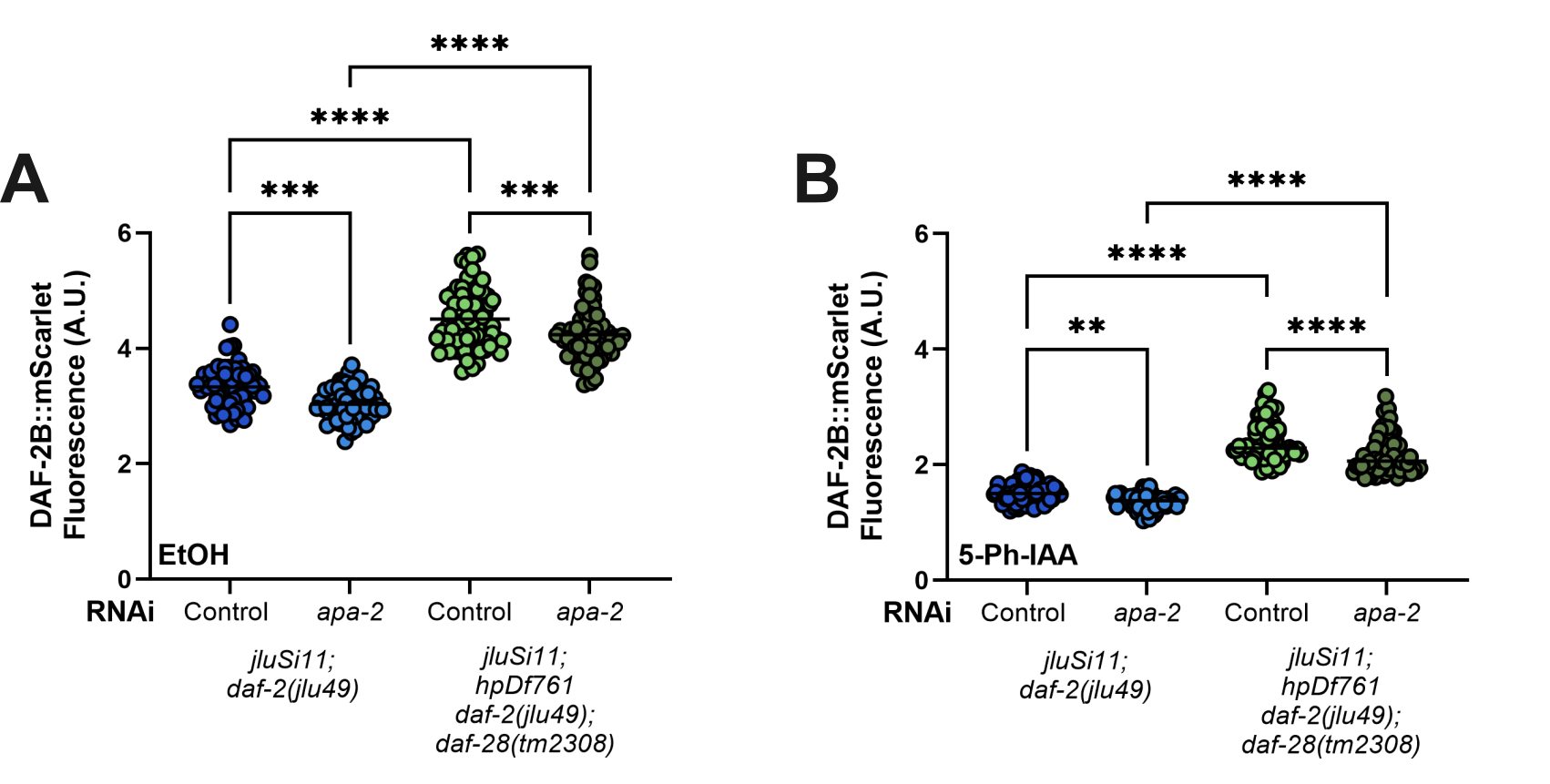

Supplement: S6 Fig — (A) Systemic apa-2 RNAi reduces expression of neuronal DAF-2B::mScarlet in jluSi11; daf-2(jlu49) and jluSi11; hpDf761; daf-2(jlu49); daf-2(tm2308) day 1 adults treated with ethanol. (B) apa-2 RNAi reduces expression of neuronal DAF-2B::mScarlet in jluSi11; daf-2(jlu49) and jluSi11; hpDf761; daf-2(jlu49); daf-2(tm2308) day 1 adults treated with 10 µM 5-Ph-IAA. hpDf761; daf-2(jlu2); daf-2(tm2308) and jluSi11; hpDf761; daf-2(jlu49); daf-2(tm2308) animals exhibited 100% dauer arrest, but spontaneously recovered within 24 h. We therefore imaged these recovered animals as D1 adults. jluSi11; daf-2(jlu49) animals did not undergo dauer arrest and were imaged as D1 adults. Data are pooled from 3 biological replicates. One-way ANOVA with Sidak’s post-hoc test for indicated pair-wise comparisons, ** p < 0.01, *** p < 0.001, **** p < 0.0001. (TIFF) [file pgen.1012240.s006.tiff]

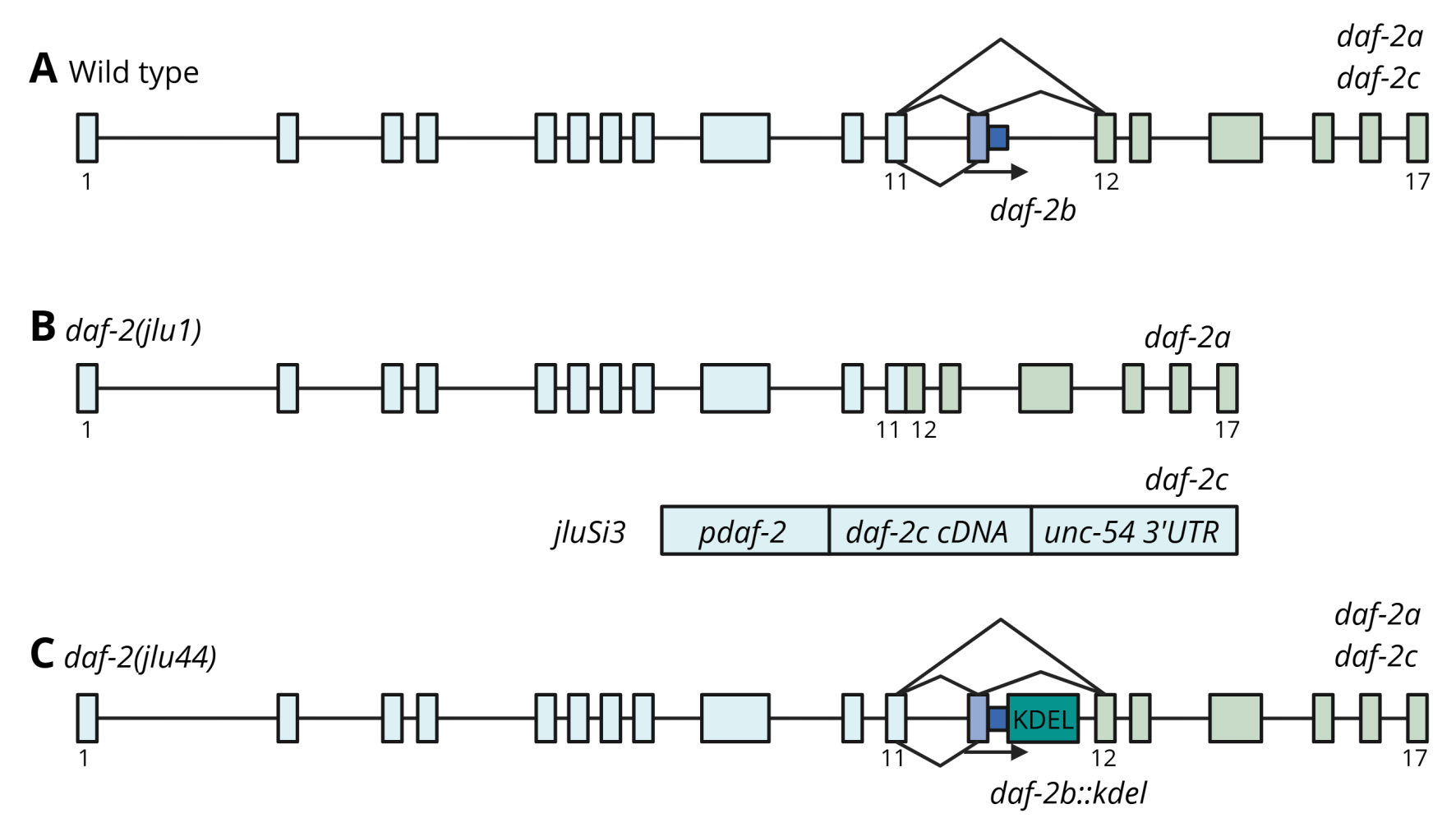

Supplement: S7 Fig — (A) Organization of the wild type daf-2 locus illustrating the splicing events that differentiate daf-2a, daf-2b and daf-2c transcripts. (B) In the daf-2b deletion mutant, jluSi3; daf-2(jlu1), the genomic sequence between exons 11 and 12 is removed, generating a daf-2b / daf-2c deletion (jlu1), with daf-2c expression restored by a single copy daf-2c cDNA insertion at the ttTi5605 safe harbor site on chromosome II (jluSi3). (C) In daf-2(jlu44), a short sequence encoding a KDEL ER retention motif has been added to the 3’ end of the daf-2b genomic locus. Created in BioRender. Gill, M. (2026) https://BioRender.com/5hynawv. (TIFF) [file pgen.1012240.s007.tiff]

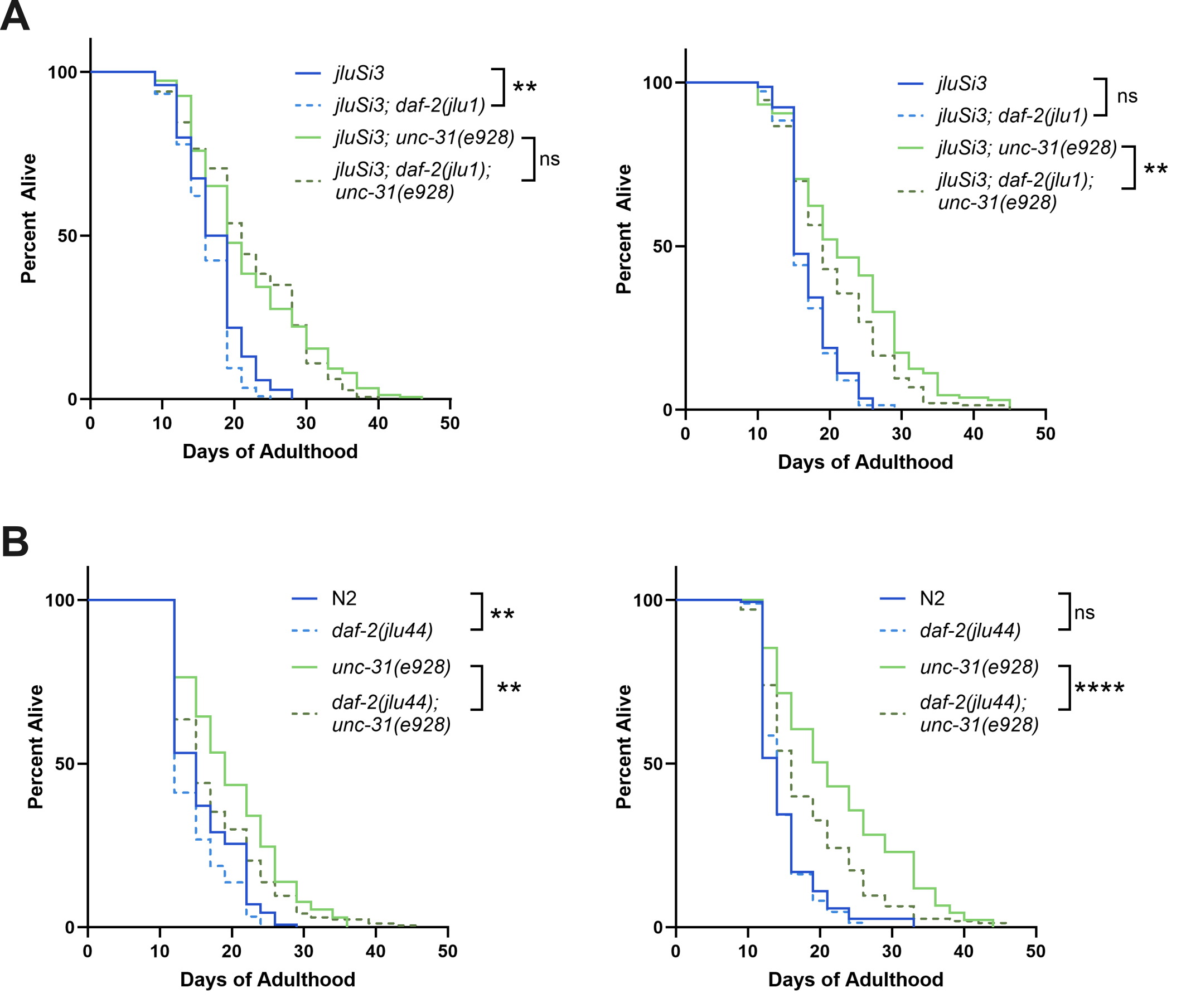

Supplement: S8 Fig — (A) Effect of jluSi3; daf-2(jlu1) on lifespan in unc-31(e928) mutants. (B) Effect of daf-2(jlu44) on lifespan in unc-31(e928) mutants. Log rank test, ns = not significant, ** p < 0.01, **** p < 0.0001 (TIFF) [file pgen.1012240.s008.tiff]

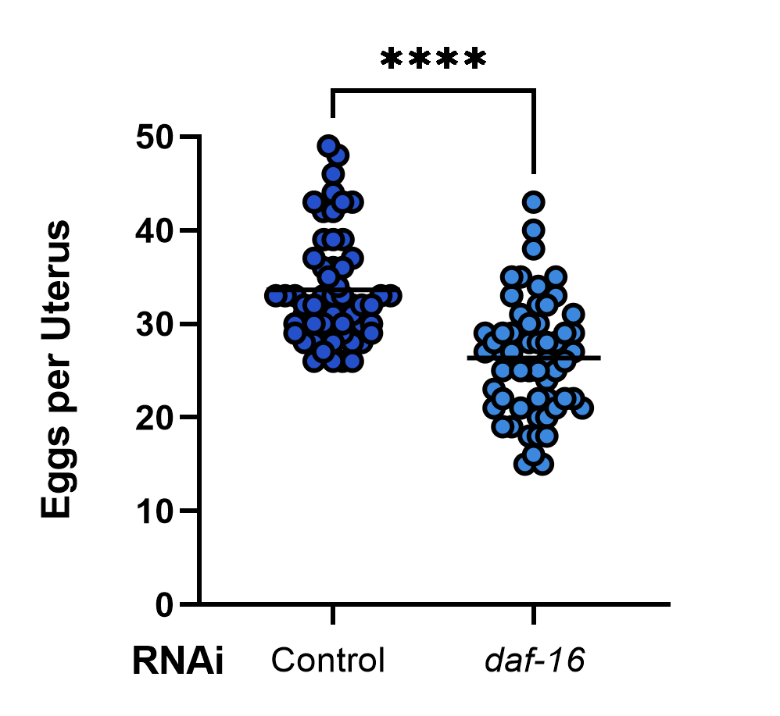

Supplement: S9 Fig — The daf-2b::KDEL mutation does not change expression of the ER stress response marker, hsp-4. qPCR was used to examine relative gene expression of hsp-4 in N2 and unc-31(e928) backgrounds with or without daf-2(jlu44). Data are derived from 6 independent populations and normalized to 2 reference genes. One-way ANOVA with Sidak’s post-hoc test for indicated pair-wise comparisons: ns = not significant. (TIFF) [file pgen.1012240.s009.tiff]

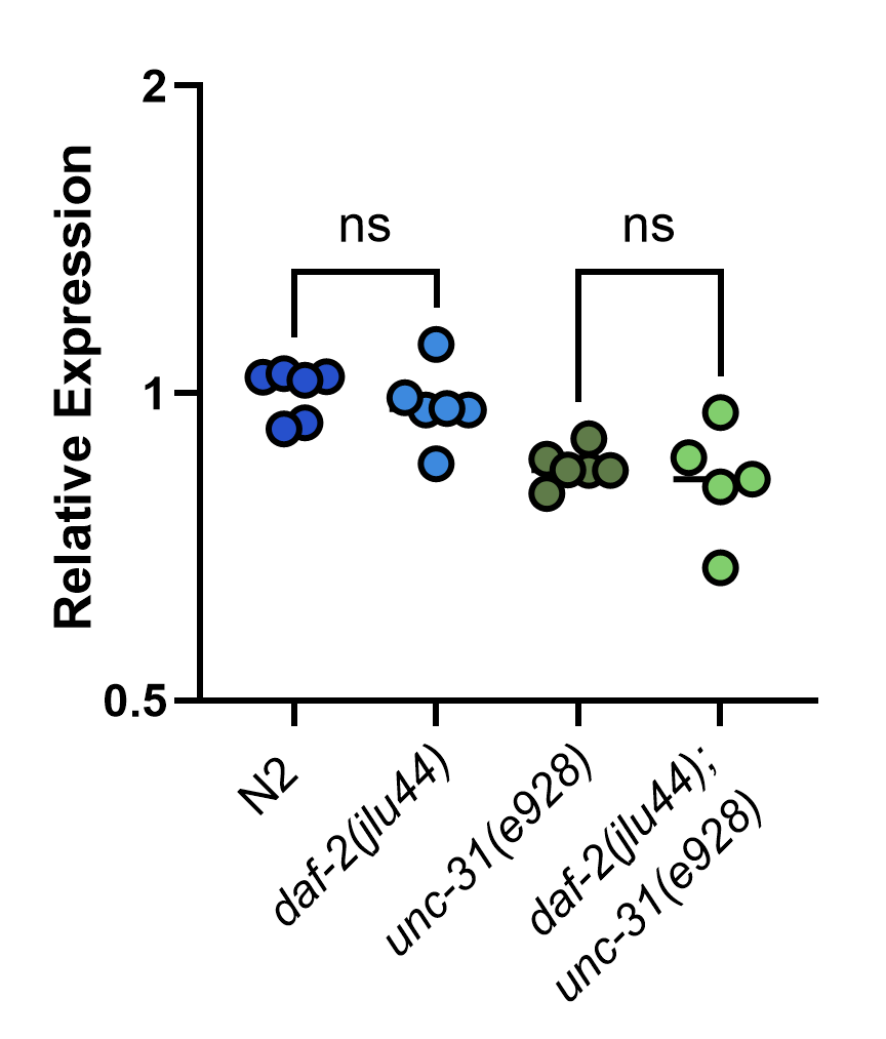

Supplement: S10 Fig — Egg retention is reduced in unc-31(e928) mutants following daf-16 RNAi. Data are pooled from 3 biological replicates. Student’s t-test: **** p < 0.0001. (TIFF) [file pgen.1012240.s010.tiff]
